# A History of Preterm Delivery Is Associated with Aberrant Postpartal MicroRNA Expression Profiles in Mothers with an Absence of Other Pregnancy-Related Complications

**DOI:** 10.3390/ijms22084033

**Published:** 2021-04-14

**Authors:** Ilona Hromadnikova, Katerina Kotlabova, Ladislav Krofta

**Affiliations:** 1 Department of Molecular Biology and Cell Pathology, Third Faculty of Medicine, Charles University, 10000 Prague, Czech Republic; katerina.kotlabova@lf3.cuni.cz; 2 Institute for the Care of the Mother and Child, Third Faculty of Medicine, Charles University, 14700 Prague, Czech Republic; ladislav.krofta@upmd.eu

**Keywords:** birth weight of newborns, cardiovascular risk, corticosteroids, expression, gestational age, microRNA, mothers, preterm prelabor rupture of membranes, spontaneous preterm birth, tocolytics, peripheral white blood cells

## Abstract

This prospective cross-sectional case-control study investigated the postpartal gene expression of microRNAs associated with diabetes/cardiovascular/cerebrovascular diseases in the peripheral white blood cells of women with anamnesis of preterm prelabor rupture of membranes (*n* = 58), spontaneous preterm birth (*n* = 55), and term delivery (*n* = 89) by a quantitative reverse transcription polymerase chain reaction. After pregnancies complicated by preterm prelabor rupture of membranes or spontaneous preterm birth, mothers showed diverse expression profiles for 25 out of 29 tested microRNAs (miR-1-3p, miR-16-5p, miR-17-5p, miR-20a-5p, miR-20b-5p, miR-21-5p, miR-23a-3p, miR-24-3p, miR-26a-5p, miR-29a-3p, miR-100-5p, miR-103a-3p, miR-125b-5p, miR-126-3p, miR-130b-3p, miR-133a-3p, miR-143-3p, miR-145-5p, miR-146a-5p, miR-181a-5p, miR-195-5p, miR-199a-5p, miR-221-3p, miR-499a-5p, and miR-574-3p). The earliest gestational ages at delivery and the lowest birth weights of newborns were associated with the highest postpartal levels of the previously mentioned microRNAs in maternal peripheral white blood cells. Administration of tocolytic drugs in order to prolong pregnancy, used in order to administer and complete a full course of antenatal corticosteroids, was associated with alterations in postpartal microRNA expression profiles to a lesser extent than in women with imminent delivery, where there was insufficient time for administration of tocolytics and antenatal corticosteroids. Overall, mothers who did not receive tocolytic therapy (miR-24-3p and miR-146a-5p) and mothers who did not receive corticosteroid therapy (miR-1-3p, miR-100-5p, and miR-143-3p) had increased or showed a trend toward increased postpartal microRNA expression when compared with mothers given tocolytic and corticosteroid therapy. In addition, mothers with serum C-reactive protein levels above 20 mg/L, who experienced preterm labour, showed a trend toward increased postpartal expression profiles of miR-143-3p and miR-199a-5p when compared with mothers with normal serum C-reactive protein levels. On the other hand, the occurrence of maternal leukocytosis, the presence of intra-amniotic inflammation (higher levels of interleukin 6 in the amniotic fluid), and the administration of antibiotics at the time of preterm delivery had no impact on postpartal microRNA expression profiles in mothers with a history of preterm delivery. Likewise, the condition of the newborns at the moment of birth, determined by Apgar scores at 5 and 10 min and the pH of cord arterial blood, had no influence on the postpartal expression profiles of mothers with a history of preterm delivery. These findings may contribute to explaining the increased cardiovascular risk in mothers with anamnesis of preterm delivery, and the greater increase of maternal cardiovascular risk with the decrease of gestational age at delivery. Women with preterm delivery in their anamnesis represent a high-risk group with special needs on a long-term basis, with a need to apply preventive and therapeutic interventions as early as possible.

## 1. Introduction

Preterm delivery in the medical history of women has been demonstrated to be associated with increased cardiovascular risk with regard to changes in body mass index (BMI) after the delivery [1], the presence of higher systolic and/or diastolic blood pressure (BP) [2,3,4,5], incident hypertension [6], chronic hypertension [1,7], systolic blood pressure associated with coronary artery calcification [8], an altered atherogenic lipid profile or hypercholesterolemia [1,2,4,7], Type 2 diabetes mellitus [1,7,9,10,11], and higher carotid intima-media thickness [2,12] in young and middle-aged women. The risk of maternal cardiovascular disease (CVD) development increases with decreasing gestational age at the time of delivery [13,14].

An initial case-control study outlined the possible association between preterm delivery of a first child and increased risk of ischaemic heart disease in mothers [15]. Recently, a large-scale national cohort study was published, which reported a 2.3% incidence of ischaemic heart disease in women, at the mean age of 57.1 years. Most ischaemic heart disease diagnoses were acute myocardial infarction (55.3%) and angina pectoris (38.7%), with other diagnoses representing the remaining proportion of acute and chronic ischaemic heart diseases [16]. Hazard ratios (HR) for the onset of ischaemic heart disease have been assessed in women with a history of preterm and term deliveries, in the 10 years after the delivery. Higher hazard ratios for later onset of ischaemic heart disease in women delivering extremely preterm (22–27 weeks, HR 4.04), very preterm (28–33 weeks, HR 2.62), and late preterm (34–36 weeks, HR 2.30)] were reported when compared with women delivering full-term [16]. Increased hazard ratios for a later onset of myocardial infarction and stroke were also reported in another independent study in women with a history of very preterm delivery (before 32 weeks gestation, HR 2.01) and moderate preterm delivery (≥32 to <37 weeks gestation, HR 1.22) [1].

In addition, preterm delivery was found to be associated with the subsequent death of mothers from both cardiovascular and non-cardiovascular causes even after exclusion of women with a history of preeclampsia (PE) and/or fetal growth restriction (FGR) [13,17,18,19,20].

Another recently published large-scale national cohort study presented data on mortality during the first 10 years after the delivery, associated with individual subcategories of preterm birth based on gestational age at delivery [21]. Increasing hazard ratios for mortality associated with declining gestational age at delivery were reported (extremely preterm delivery (22–27 weeks, HR 2.28), very preterm delivery (28–33 weeks, HR 1.52), and late preterm delivery (34–36 weeks, HR 1.19)) [21].

Moreover, stronger associations between elective preterm delivery (induced vaginal birth or Caesarean section (CS)) and ischaemic heart disease events and ischaemic heart disease death were observed when compared with spontaneous preterm delivery [16,22].

Overall, a meta-analysis of 21 studies involving 338,000 women with a history of preterm delivery demonstrated higher risk ratios (RR) for later onset of any cardiovascular disease (RR 1.43), coronary heart disease (RR 1.49), and stroke (RR 1.65). Moreover, higher risk ratios for cardiovascular disease-associated death (RR 1.78) and coronary heart disease-associated death (RR 2.10) were reported in women with a history of preterm delivery [23].

Women with preterm delivery in anamnesis have also been shown to have an increased risk of subsequent ophthalmic complications, such as diabetic retinopathy and glaucoma [24], chronic kidney disease, end-stage kidney disease [25], and breast cancer [26,27].

Recently, we reported that a proportion of mothers previously affected by hypertensive and metabolic disorders during pregnancy, and placental insufficiency-related complications, had altered postpartal microRNA expression profiles in their peripheral white blood cells (WBC) on a long-term basis, which may contribute alongside other factors to the development of diabetes mellitus and cardiovascular and cerebrovascular diseases [28,29].

MicroRNAs, small single-stranded non-coding RNA molecules [30,31,32,33], regulate many diverse processes, including cardiovascular system development and functioning, and offer novel perspectives as potential diagnostic and prognostic biomarkers and therapeutic targets in multiple conditions, ranging from cardiovascular risk factors (hypertension, diabetes mellitus, dyslipidaemia, atherosclerosis, etc.) to various cardiovascular and cerebrovascular pathologies, such as congenital heart diseases, arrhythmia, coronary artery disease, myocardial infarction, heart failure, atrial fibrillation, cardiac hypertrophy, fibrosis, and cerebral ischemic events [34,35,36,37,38,39,40] (Table S1). MicroRNAs regulate gene expression at the post-transcriptional level by blocking translation or degradation of target messenger RNA [30].

The main goal of the current study was to determine to what extent alterations in the gene expression of microRNAs associated with diabetes and cardiovascular/cerebrovascular diseases are present 3–11 years postpartum in the peripheral white blood cells of young and middle-aged women with a history of preterm delivery (preterm prelabor rupture of membranes (PPROM) and spontaneous preterm birth (PTB)), excluding cases of gestational diabetes mellitus (GDM), gestational hypertension (GH), preeclampsia, fetal growth restriction, and other pregnancy-related complications (placenta previa, placental abruption, and vaginal bleeding).

We were also interested in the global impact of multiple factors on postpartal microRNA expression profiles in women with a history of preterm delivery. These factors included prenatal clinical findings (maternal leukocytosis, increased maternal serum C-reactive protein (CRP) levels, signs of intra-amniotic inflammation), applied therapies (corticosteroid therapy, antibiotic therapy, and tocolytic therapy), the course of the delivery (the type of premature delivery, the subcategories of preterm birth based on gestational age, and the mode of delivery), and clinical parameters of the newborns (the birth weight and the condition of infants at the moment of birth determined using the Apgar Scores (AS) and pH of cord arterial blood).

CRP, which is a sensitive inflammatory serum marker that reflects infectious and inflammatory processes and can predict preterm labor and response to tocolytic therapy in pregnant women [41,42,43,44,45,46,47,48], was involved in the analyses since it represents a risk indicator for developing cardiovascular problems [49,50,51,52,53,54,55,56,57,58,59,60]. Similarly, interleukin 6 (IL-6), an inflammatory cytokine and an indicator of preterm birth and intra-amniotic inflammation [61,62], was also involved in the analyses, since IL-6 is a major player in the pathogenesis of atherosclerosis, and its blockade may reduce cardiovascular risk in high-risk populations [63]. Previously, it was reported that the mode of delivery and presence/absence of labor may result in expression differences of some microRNAs in women with preeclampsia [64]. Therefore, we also investigated the impact of the mode of delivery on postpartal microRNA gene expression in women with anamnesis of preterm delivery.

To the best of our knowledge, there are currently no data on postpartal expression profiles of microRNAs associated with diabetes mellitus and cardiovascular and cerebrovascular diseases in women with anamnesis of preterm delivery, in the absence of other pregnancy-related complications. In addition, there are no data on the long-term impact of corticosteroid, tocolytic, and antibiotic therapies administered to pregnant women at risk of preterm delivery on postpartal microRNA expression profiles.

An investigation into the early and late effects of prenatal corticosteroid treatment on the profiles of certain microRNAs has been previously performed, but in the lung tissue of experimental animal models only [65].

A set of 29 microRNAs known to be involved in the pathogenesis of diabetes mellitus and cardiovascular and cerebrovascular diseases was selected for our previous and currently ongoing studies (Table S1).

## 2. Results

### 2.1. Clinical Outcomes in Mothers with a History of Preterm Delivery

The anamnesis of patients, the course of gestation, and the delivery type were obtained from the medical records of patients during gestation and delivery. Furthermore, during the follow-up study (held 3–11 years postpartum), additional clinical data of patients (including data concerning the incidence of cardiovascular risk factors and the incidence of cardiovascular and cerebrovascular diseases) were collected by our team during clinical examinations of invited patients who had delivered in our institution. This follow-up study was very valuable since many cardiovascular risk factors had not been present in these patients during the pregnancy course and during admission to delivery. Many cardiovascular risk factors appeared, evidently, after a certain time after the delivery, and were identified during our follow-up study.

Although there were not enough patients in each group to determine the statistical difference between women with a history of preterm deliveries and those who had at-term deliveries, it was clear that some factors, which increase cardiovascular risk (birth defects of the heart, heart arrhythmia, and rheumatoid arthritis) appeared more frequently in the group of women with anamnesis of preterm delivery.

Moreover, some severe cardiovascular and cerebrovascular events (pulmonary embolism or recurrent cerebrovascular accidents) had already appeared in two women with anamnesis of preterm delivery in young and middle ages.

The incidence of trombophilic gene mutations was significantly higher in women with a history of either PPROM or PTB despite the low number of patients who experienced this in each group.

In addition, a trend toward a higher BMI and diastolic BP (DBP) values was observed in women with anamnesis of PPROM, when compared with women with anamnesis of normal term delivery.

Furthermore, a higher incidence of infertility treatment was observed in women with a history of either PPROM or PTB when compared with women with a history of normal term delivery (Table 1).

### 2.2. Substantially Altered Postpartal Expression Profiles in the Peripheral White Blood Cells of Mothers with a History of Preterm Delivery

Initially, we compared postpartal microRNA gene expression profiles in the peripheral white blood cells of mothers with a history of preterm prelabor rupture of membranes, spontaneous preterm birth, and normal course of gestation.

Both pilot and validation studies in two independent cohorts of patients were performed. Sample size calculation was used to calculate the minimal required sample size of subjects for these analyses. Both pilot and validation studies on minimal sample sizes of subjects showed higher postpartal levels of the most tested microRNAs in women with anamnesis of preterm delivery (PPROM or PTB) (Table 2).

Consecutive experimental data obtained from a larger independent cohort of patients with a history of PPROM (*n* = 58) and PTB (*n* = 55) are reported below in detail. Significantly increased postpartal expression of most microRNAs (miR-1-3p, miR-16-5p, miR-17-5p, miR-20a-5p, miR-20b-5p, miR-21-5p, miR-23a-3p, miR-24-3p, miR-26a-5p, miR-29a-3p, miR-100-5p, miR-103a-3p, miR-125b-5p, miR-126-3p, miR-130b-3p, miR-133a-3p, miR-143-3p, miR-145-5p, miR-146a-5p, miR-181a-5p, miR-195-5p, miR-199a-5p, miR-221-3p, miR-499a-5p, and miR-574-3p) was detected in mothers with a history of either PPROM or PTB after Benjamini-Hochberg correction for multiple comparisons, which itself was applied after the Kruskal-Wallis test (Table 3a) (Figure S1). An aberrant microRNA expression profile was able to differentiate between mothers after normal pregnancies and mothers after pregnancies complicated with PPROM or PTB. These findings may indicate an increased risk of later development of diabetes and/or cardiovascular/cerebrovascular diseases in women with a history of either PPROM or PTB.

In Table 3a, a Benjamini-Hochberg correction for multiple comparisons shows a comparison of postpartal microRNA gene expression between women with a history of preterm and term deliveries (normal pregnancies, PPROM, and PTB). 

### 2.3. The Association between Gestational Age at Delivery and Postpartal Expression of Diabetes/Cardiovascular/Cerebrovascular Disease-Associated MicroRNAs

After Benjamini-Hochberg correction for multiple comparisons was applied after the Kruskal-Wallis test (Table 3b), women with a history of preterm delivery in all individual subcategories of preterm birth, based on gestational age (extremely preterm, very preterm, and moderate to late preterm), showed significantly increased postpartal microRNA gene expression (miR-1-3p, miR-20a-5p, miR-20b-5p, miR-26a-5p, miR-29a-3p, miR-100-5p, miR-103a-3p, miR-125b-5p, miR-126-3p, miR-130b-3p, miR-133a-3p, miR-143-3p, miR-145-5p, miR-181a-5p, miR-195-5p, miR-199a-5p, and miR-499a-5p) in the peripheral white blood cells, when compared to women with a history of normal pregnancies (Figure S2).

In addition, mothers with a history of very preterm birth and moderate to late preterm birth had substantially increased postpartal expression of miR-16-5p, miR-17-5p, miR-21-5p, miR-23a-3p, miR-24-3p, miR-146a-5p, miR-221-3p, and miR-574-3p. Mothers with a history of moderate to late preterm birth had also substantially increased postpartal expression of miR-342-3p (Figure S2).

The postpartal microRNA expression profile did not differ between women with a history of preterm delivery divided into individual subcategories of preterm birth based on gestational age (extremely preterm, very preterm, and moderate to late preterm) (Figure S2).

In Table 3b, the Benjamini-Hochberg correction for multiple comparisons shows a comparison of postpartal microRNA gene expression between women with a history of preterm and term deliveries, based on gestational age (normal pregnancies, extremely preterm birth, very preterm birth, and moderate to late preterm birth).

Nevertheless, in the total group of women, including both those with a history of normal and complicated pregnancies, a strong negative correlation between postpartal microRNA gene expression (miR-1-3p, miR-103a-3p, miR-126-3p, miR-143-3p, miR-181a-5p, and miR-499a-5p) in the peripheral white blood cells of mothers and the gestational age at delivery was observed. In addition, a weak negative correlation between postpartal microRNA gene expression (miR-16-5p, miR-17-5p, miR-20a-5p, miR-20b-5p, miR-21-5p, miR-23a-3p, miR-24-3p, miR-26a-5p, miR-29a-3p, miR-92a-3p, miR-100-5p, miR-125b-5p, miR-130b-3p, miR-133a-3p, miR-145-5p, miR-146a-5p, miR-195-5p, miR-199a-5p, miR-221-3p, miR-342-3p, and miR-574-3p) in the peripheral white blood cells of mothers and the gestational age at delivery was found (Table 4 and Figure S3). This finding indicates that women delivering at term have significantly lower postpartal levels of diabetes/cardiovascular/cerebrovascular disease-associated microRNAs than women with a history of preterm delivery (PPROM or PTB). Once again, this finding may support the fact that women with a history of either PPROM or PTB have an increased risk of later development of diabetes and/or cardiovascular/cerebrovascular diseases.

### 2.4. The Association between Birth Weight of Newborns and Postpartal Expression of Diabetes/Cardiovascular/Cerebrovascular Disease-Associated MicroRNAs

In parallel, in the total group of women (women after both normal and complicated pregnancies), a strong negative correlation between postpartal microRNA gene expression (miR-1-3p, miR-17-5p, miR-20b-5p, miR-26a-5p, miR-29a-3p, miR-100-5p, miR-103a-3p, miR-126-3p, miR-130b-3p, miR-143-3p, miR-181a-5p, miR-199a-5p, and miR-499a-5p) in the peripheral white blood cells of mothers and the birth weight of the newborns was observed. A weak negative correlation between postpartal microRNA gene expression (miR-16-5p, miR-20a-5p, miR-21-5p, miR-23a-3p, miR-24-3p, miR-92a-3p, miR-125b-5p, miR-133a-3p, miR-145-5p, miR-146a-5p, miR-195-5p, miR-221-3p, miR-342-3p, and miR-574-3p) in the peripheral white blood cells of mothers and the birth weight of the newborns was also found (Table 5 and Figure S4). This finding indicates that women delivering full-term infants with normal birth weight have significantly lower postpartal levels of diabetes/cardiovascular/cerebrovascular disease-associated microRNAs than those delivering premature low birth weight infants. Once more, this finding may contribute to explaining why women with obstetrical anamnesis of preterm delivery in their history have an increased cardiovascular risk.

### 2.5. Association between the Mode of Delivery and Postpartal Expression of Diabetes/Cardiovascular/Cerebrovascular Disease-Associated MicroRNAs in Maternal Peripheral White Blood Cells

No association between the mode of delivery (vaginal birth or caesarean birth) and postpartal expression of diabetes/cardiovascular/cerebrovascular disease-associated microRNAs in the peripheral white blood cells was observed in mothers with a history of PPROM or PTB.

### 2.6. Association between Maternal Serum C-Reactive Protein Levels during Previous Gestation and Postpartal Expression of Diabetes/Cardiovascular/Cerebrovascular Disease-Associated MicroRNAs in Peripheral White Blood Cells

A trend toward increased postpartal gene expression of miR-143-3p (*p* = 0.090) and miR-199a-5p (*p* = 0.055) was detected in women with a history of preterm delivery, with serum CRP levels above 20 mg/L in preterm labour (Figure S5).

Nevertheless, no correlation between maternal serum CRP levels during previous gestation and postpartal expression of diabetes/cardiovascular/cerebrovascular disease-associated microRNAs in the peripheral white blood cells was observed in mothers with a history of preterm delivery.

Since no correlation between antenatal maternal serum CRP levels and postpartal microRNA gene expression was observed, these microRNA biomarkers may have limited significance for differentiation between women with a history of preterm delivery, with regard to low and high cardiovascular risk.

### 2.7. Association between Maternal Leukocytosis and Interleukin 6 Levels in the Amniotic Fluid during Previous Gestation and Postpartal Expression of Diabetes/Cardiovascular/Cerebrovascular Disease-Associated MicroRNAs in Peripheral White Blood Cells

No association between maternal leukocytosis (total white blood cell count above the normal) during previous gestation and postpartal expression of diabetes/cardiovascular/cerebrovascular disease-associated microRNAs in the peripheral white blood cells was observed in mothers with a history of PPROM or PTB. In addition, no association between signs of intra-amniotic inflammation during previous gestation (levels of IL-6 in the amniotic fluid) and postpartal microRNA gene expression in the peripheral white blood cells was found. It should be noted that only a few patients had their IL-6 levels in the amniotic fluid measured for this study.

### 2.8. Association between the Application of Corticosteroid, Antibiotic, and Tocolytic Therapies during Previous Gestation and Postpartal Expression of Diabetes/Cardiovascular/Cerebrovascular Disease-Associated MicroRNAs in Peripheral White Blood Cells

Women treated with corticosteroids during previous gestation to accelerate lung maturation in the fetus had lower postpartal expression levels of miR-1-3p (*p* = 0.027) when compared with women who did not receive corticosteroid therapy, who usually delivered immediately after admission to hospital. In addition, the postpartal levels of miR-100-5p (*p* = 0.066) and miR-143-3p (*p* = 0.070) showed a trend toward increased expression in mothers who did not receive corticosteroid therapy during their previous gestation (Figure S6).

In parallel, women treated with tocolytics to postpone premature labor also showed a trend toward decreased postpartal expression levels of miR-24-3p (*p* = 0.071) and miR-146a-5p (*p* = 0.053) when compared with women not given tocolysis, when postponing the delivery long enough for the administration of glucocorticoids was impossible (Figure S7).

On the other hand, no association was observed between antibiotic administration for women in preterm labour and postpartal expression of diabetes/cardiovascular/cerebrovascular disease-associated microRNAs in the peripheral white blood cells.

### 2.9. Association between the Condition of Newborns at the Moment of Birth and Postpartal Expression of Diabetes/Cardiovascular/Cerebrovascular Disease-Associated MicroRNAs in Peripheral White Blood Cells

No association between Apgar scores, determined at 5 min and 10 min after birth, of the newborns, and postpartal expression of diabetes/cardiovascular/cerebrovascular disease-associated microRNAs in the peripheral white blood cells, was observed in mothers with a history of PPROM or PTB. In addition, no association between a parameter of metabolic acidosis in the newborns (pH of cord arterial blood) and postpartal microRNA gene expression in the peripheral white blood cells was found.

### 2.10. Information on MicroRNA-Gene-Biological Pathways/Disease Interactions

Information on microRNA-gene-biological pathways/disease interactions was provided on microRNAs dysregulated in the peripheral white blood cells of women with a history of preterm delivery (PPROM and/or PTB). Predicted targets of microRNAs involved in key human biological pathways, with a role in the pathogenesis of preterm delivery, have been reported. These biological pathways involve apoptosis, inflammatory response, senescence, and autophagy. In addition, predicted targets of microRNAs associated with cardiovascular risk factors (infertility, thrombophilia, higher values of BMI, and diastolic blood pressure (DBP)) that appeared more frequently in our group of women with a history of PPROM and/or PTB were also reported.

A large group of genes (predicted targets) of cardiovascular and/or cerebrovascular disease-associated microRNAs aberrantly expressed in the peripheral white blood cells of women with a history of PPROM and/or PTB is also involved in key biological processes related to pathogenesis of preterm delivery, such as the apoptosis pathway, an inflammatory response pathway, and senescence and autophagy pathways (Table 6, Table 7 and Table 8).

In addition, predicted targets of microRNAs aberrantly expressed in the peripheral white blood cells of mothers with a history of PPROM and/or PTB indicated that a large group of genes is also involved in the pathogenesis of diseases, which increase cardiovascular risk in women with a history of PPROM and/or PTB (infertility, thrombophilia, susceptibility to obesity, and hypertension) (Table 9, Table 10, Table 11 and Table 12).

## 3. Discussion

We compared postpartal microRNA expression profiles in the peripheral white blood cells between women with a history of pregnancies complicated by preterm delivery, and women with a history of normal pregnancies. The group of complicated pregnancies consisted of women with a history of preterm prelabor rupture of membranes and spontaneous preterm birth. Women with the presence of other coincident pregnancy-related complications were intentionally excluded from the study since postpartal microRNA expression profiles have already been studied separately in some pregnancy-related complications (gestational hypertension, preeclampsia, gestational diabetes mellitus, and fetal growth restriction) [28,29]. Women with a history of preterm delivery complicated by placenta previa, placental abruption, and significant vaginal bleeding were also excluded from participation in the study, with the aim of studying a homogenous group of patients.

In total, significantly diverse microRNA expression profiles were detected in mothers after pregnancies complicated with either PPROM or PTB.

Almost all examined microRNAs (miR-1-3p, miR-16-5p, miR-17-5p, miR-20a-5p, miR-20b-5p, miR-21-5p, miR-23a-3p, miR-24-3p, miR-26a-5p, miR-29a-3p, miR-100-5p, miR-103a-3p, miR-125b-5p, miR-126-3p, miR-130b-3p, miR-133a-3p, miR-143-3p, miR-145-5p, miR-146a-5p, miR-181a-5p, miR-195-5p, miR-199a-5p, miR-221-3p, miR-499a-5p, and miR-574-3p) showed increased expression in the peripheral white blood cells of mothers with a history of preterm delivery in their anamnesis. Next to mothers with a history of GDM [29], this is the most serious finding we have observed during examination of postpartal expression profiles of microRNAs associated with diabetes mellitus and cardiovascular and cerebrovascular diseases in groups of women with a history of pregnancy-related complications [28,29].

The presence of substantially altered postpartal microRNA expression profiles in young and middle-aged women with a history of preterm delivery may partially explain the higher incidence of CVD risk factors, CVD events (myocardial infarction, stroke, angina pectoris, other acute and chronic ischemic heart disease diagnoses), and CVD-associated mortality [1,2,3,4,5,6,7,8,9,10,11,12,13,14,15,16,17,18,19,20,21,22,23].

For this reason, we strongly support the suggestion of other investigators that preterm delivery represents a strong independent predictor of later onset of CVD, and that women with preterm delivery in their anamnesis represent a high risk group with special needs on a long-term basis, in terms of a need to apply preventive and therapeutic interventions as early as possible [1,2,3,4,5,6,7,8,9,10,11,12,13,14,15,16,17,18,19,20,21,22,23,66].

Subsequently, we analysed microRNA expression profiles in the peripheral white blood cells of mothers with preterm delivery in relation to the gestational age at delivery and the birth weight of the newborns. It was evident that both the earliest gestational ages at delivery and the lowest birth weights of newborns, which are usually a result of early gestational age at delivery, were associated with the highest postpartal levels of microRNAs in the peripheral white blood cells, which may also explain the increased cardiovascular risk in mothers with anamnesis of preterm delivery [1,16,21,66], and the increase of maternal cardiovascular risk with the decrease of gestational age at delivery [13,14].

Similarly, administration of tocolytic drugs in order to prolong pregnancy for long enough to start and complete a course of antenatal corticosteroids was associated with alterations in microRNA expression profiles to a lesser extent than in women who experienced imminent delivery, for whom there was insufficient time for administration of tocolytics and antenatal corticosteroids. A trend toward higher postpartal levels of miR-24-3p and miR-146a-5p was found in women who were not administered tocolytics, when compared with women who underwent tocolytic therapy. Higher postpartal levels of miR-1-3p and a trend toward higher postpartal levels of miR-100-5p and miR-143-3p were observed in women who were not administered antenatal corticosteroids, when compared with women given antenatal corticosteroids. This finding again may support the fact that maternal cardiovascular risk is increased with the decrease of gestational age at delivery [13,14], since the administration of tocolytic drugs prolongs pregnancy and enables the administration of corticosteroids to accelerate fetal lung maturation.

In addition, C-reactive protein, a nonspecific biomarker of inflammation, is associated with a large number of medical conditions inclusive of preterm delivery [41,42,43,44,45,46,47,48], and had a partial impact on the postpartal microRNA expression profiles of women with a history of preterm delivery. Women with increased serum CRP levels in terms of preterm labour displayed a trend toward postpartal upregulation of miR-143-3p and miR-199a-5p in the peripheral white blood cells. However, this finding may have limited significance for differentiation between women with a history of preterm delivery with regard to low and high cardiovascular risk, since no correlation between antenatal maternal serum CRP levels and postpartal microRNA gene expression was observed.

On the other hand, the occurrence of leukocytosis in maternal peripheral blood and the presence of intra-amniotic inflammation (higher levels of IL-6 in the amniotic fluid), and the administration of antibiotics at the time of preterm delivery, had no impact on postpartal microRNA expression profiles in mothers with a history of preterm delivery. Likewise, the condition of newborns at the moment of birth, determined by Apgar scores at 5 and 10 min after birth and the pH of cord arterial blood, had no influence on the postpartal expression profiles of mothers with a history of preterm delivery.

This study has some limitations. A set of 29 microRNAs, known to be involved in pathogenesis of diabetes mellitus, cardiovascular disease, and cerebrovascular diseases, was selected for our previous and currently ongoing studies (Table S1) [28,29]. However, the list of selected microRNAs did not include all the microRNAs known to be involved in the pathogenesis of diabetes mellitus and cardiovascular and cerebrovascular diseases. In addition, the list of selected microRNAs for our previous and currently ongoing studies [28,29] was not based on genome-wide expression studies since these analyses had not been performed in the cohorts of our patients.

In addition, another possible limitation of this study is the fact that, while dysregulated microRNAs play a role in the functioning of metabolism and the cardiovascular system, there are certainly other risk factors that can contribute to the development of diabetes mellitus and cardiovascular and cerebrovascular diseases.

Nevertheless, it is clear that some factors, which increase cardiovascular risk (birth defects of the heart, heart arrhythmia, and rheumatoid arthritis) appeared more frequently in our group of young and middle-aged women with anamnesis of preterm delivery. Moreover, some severe cardiovascular and cerebrovascular events had already occurred in our group of women with anamnesis of preterm delivery in the young and middle ages. On the other hand, pulmonary embolism or recurrent cerebrovascular accidents had been diagnosed in just two women with a history of preterm delivery.

The incidence of infertility and trombophilic gene mutations was significantly higher in our group of women with a history of either PPROM or PTB. In addition, a trend toward higher BMI and DBP values was observed in the group of women with anamnesis of PPROM when compared with women with anamnesis of normal term delivery.

On the basis of these data, we can hypothesize that altered microRNA expression profiles in whole peripheral white blood cells of young and middle-aged women with a history of preterm delivery are associated with increased cardiovascular risk. Nevertheless, further studies are needed to evaluate the possible role of postpartal microRNA expression as a predictor for later development of diabetes mellitus and cardiovascular and cerebrovascular diseases. We realize that much longer follow-up of patients is needed to observe the capability of altered microRNA expression profiles in young and middle-aged women to predict the onset of cardiovascular and cerebrovascular diseases later in their life.

We assume that the long-term dysregulation of cardiovascular and cerebrovascular disease-associated microRNAs in women with a history of preterm delivery might be induced by a pathological course of gestation itself. The aetiology of preterm delivery is multifactorial, and remains unknown in most cases. Nevertheless, many possible pathogenic mechanisms have been identified. These include an incomplete cervix, uterine abnormalities, intraamniotic infection with activation of the innate immune system, and an exaggeration of inflammatory processes [67,68,69,70,71,72,73]. Furthermore, key biological pathways, such as premature aging of the fetal membranes, where senescence, apoptosis, and proteolysis play an important role, have been identified in women with preterm delivery [74,75,76]. Interactions between cardiovascular and cerebrovascular disease-associated microRNAs dysregulated in the peripheral white blood cells of women with anamnesis of PPROM and/or PTB and specific genes involved in key biological processes related to pathogenesis of preterm delivery, such as the apoptosis pathway, inflammatory response pathway, and senescence and autophagy pathways, were documented in this study.

Alternatively, aberrant microRNA expression profiles might have already been present in these women before gestation, and might predispose them to a complicated course of gestation in the form of preterm delivery.

In addition, some diseases which increase cardiovascular risk (infertility and the presence of trombophilic gene mutations), together with therapeutic approaches (infertility treatment and antithrombotic prophylaxis applied to carriers of trombophilic gene mutations), might also contribute to modifications of microRNA expression profiles in women with a history of preterm delivery.

Furthermore, some abnormal clinical findings, which contribute to the increase of cardiovascular risk (a trend towards higher BMI and DBP values) might induce novel alterations or intensify present alterations in microRNA gene expression profiles in women with anamnesis of PPROM. Interactions between microRNAs dysregulated in the peripheral blood white blood cells of women with anamnesis of PPROM and/or PTB and specific genes related to human disease ontologies, such as infertility, thrombophilia, susceptibility to hypertension, and obesity, were also documented in this study.

## 4. Materials and Methods

### 4.1. Participants

The prospective cross-sectional case-control study included Caucasian women with a history of normal singleton pregnancies (*n* = 89) and singleton pregnancies complicated with preterm prelabor rupture of membranes (*n* = 58) or spontaneous preterm birth (*n* = 55). The clinical data of participants are presented in Table 1.

Women with a history of normal pregnancies were defined as those with an absence of medical, obstetrical, or surgical complications, giving birth to healthy infants weighing more than 2500 g, after 37 completed weeks of gestation.

The occurrence of regular uterine contractions at a minimum frequency of two contractions per 10 min, along with cervical changes, leading to delivery before the 37th week of gestation, was defined as PTB. Amniotic fluid leakage preceding the onset of labour by at least 2 h was defined as PPROM [77,78,79].

Women with signs of gestational hypertension, preeclampsia, gestational diabetes mellitus, and fetal growth restriction were not included in the study. Women with a history of pregnancies complicated with inborn defects and chromosomal abnormalities in the foetus or the newborn, as well as women with a history of pregnancies affected by other complications (placenta previa, placental abruption, and significant vaginal bleeding), were excluded from participation in the study.

Twelve women at risk of preterm birth had maternal serum CRP levels above the 95th percentile (>20 mg/L) [80]. Sixteen women at risk of preterm birth had maternal leukocytosis (serum white blood cell (WBC) levels above 16.9 × 10^9^/L) [80]. Intra-amniotic inflammation was diagnosed when the IL-6 amniotic fluid concentration was >2.6 ng/mL [81]. IL-6 was assessed in only five patients at risk of preterm birth. Three out of these five women had significantly higher levels of IL-6 in the amniotic fluid, ranging from 5.0 to 96.46 ng/mL.

Antenatal corticosteroids for accelerating fetal lung maturation were given to pregnant women between 24 0/7 weeks and 33 6/7 weeks of gestation, who were at risk of preterm delivery within 7 days [81,82,83]. In addition, antenatal corticosteroids were administered to pregnant women between 34 0/7 weeks and 36 6/7 weeks of gestation who were at risk of preterm delivery within 7 days, and who had not received a previous course of antenatal corticosteroids [82,83,84]. Altogether, in our cohort, 84 out of 113 women were treated with corticosteroids.

Prophylactic antibiotic therapy was administrated among the majority of women in preterm labour (*n* = 90), with the exception of women with intact amniotic membranes and no clinical signs of infection (*n* = 23) [85,86].

The use of tocolytics was individualized, and tocolytics were not used when there was any obstetric or medical contraindication for prolonging the pregnancy [86,87,88].

In total, tocolytic therapy was applied to 66 out of 113 tested women at risk of imminent preterm birth, who had experienced an otherwise uncomplicated pregnancy, with the aim to prolong the pregnancy to provide a window for administration of antenatal corticosteroids and/or in-utero fetal transfer to an appropriate neonatal healthcare setting.

Concerning subcategories of preterm birth based on gestational age [89,90,91], 19 women delivered extremely preterm infants (less than 28 weeks), 33 women delivered very preterm infants (28 to 32 weeks), and 61 women delivered moderate to late preterm infants (32 to 37 weeks).

A caesarean section was performed for obstetric indications in 48 out of 113 examined women in refractory preterm labour [92,93,94].

The Apgar scores, which indicate the health status of the newborn infant immediately after birth and the response to resuscitation if needed, were determined at 1 min, 5 min, and 10 min after birth [95,96,97,98]. A score of 7 to 10 (reassuring) after five minutes was detected in 105 preterm newborns. A score of 4 to 6 (moderately abnormal) was detected in five preterm newborns, and a score of 0 to 3 (concerning), indicating a need for increased intervention, was detected in three preterm newborns.

Umbilical cord blood analysis, which gives the acid–base balance of the infant at the moment of birth, was also performed. Significant metabolic acidosis was defined as cord arterial blood pH < 7.0, and was detected in 8 out of 113 preterm infants.

Informed consent was signed by all participants involved in the study. The Ethics Committee of the Institute for the Care of the Mother and Child and The Ethics Committee of the Third Faculty of Medicine, Charles University, approved the study (grant no. AZV 16-27761A, long-term monitoring of complex cardiovascular profiles in the mother, fetus, and offspring descending from pregnancy-related complications, dates of approval: 27.3.2014 and 28.5.2015). All procedures were in compliance with the Helsinki Declaration of 1975, as revised in 2000.

### 4.2. Sample Processing, Reverse Transcription, and Relative MicroRNA Quantification

Sample processing, reverse transcription, and relative microRNA quantification in the peripheral white blood cells of mothers were undertaken as previously described [28,29,99,100]. A detailed description of these procedures is also available in the Supplementary Materials.

### 4.3. Data Processing

Statistical analyses (the Shapiro-Wilk test, Mann-Whitney test (M-W), Kruskal-Wallis test (K-W), and the Spearman rank correlation coefficient (ρ)) were carried out, as has already been described [28,29,101]. The Benjamini-Hochberg correction, controlling for the false discovery rate (FDR) using a sequential modified Bonferroni correction for multiple comparisons, was applied after the Kruskal-Wallis test to set up new cut-off values and interpret the experimental data.

Box plots representing log-normalized expression values (quantitative reverse transcription polymerase chain reaction (RT-qPCR) expression, log_10_ 2^−ΔΔCt^) for individual microRNAs are presented. Detailed descriptions of the statistical and graphical processing can be found in the Supplementary Materials.

### 4.4. Information on MicroRNA-Gene-Biological Pathways/Disease Interactions

The MiRWalk database (available: http://www.umm.uni-heidelberg.de/apps/zmf/mirwalk/ (accessed on 31 March 2021)) and the Predicted Target module were used to provide information on predicted targets of microRNAs dysregulated in the peripheral white blood cells of women with a history of preterm delivery (PPROM and/or PTB) [102].

MiRWalk is a comprehensive database that provides information on microRNAs from humans, mice, and rats on their predicted and/or validated target genes. miRWalk2.0 not only documents miRNA binding sites within the complete sequence of a gene, but also combines this information with a comparison of binding sites from 12 existing miRNA-target prediction programs (DIANA-microTv4.0, DIANA-microT-CDS, miRanda, mirBridge, miRDB4.0, miRmap, miRNAMap, DoRiNA, PicTar2, PITA, RNA22v2, RNAhybrid2.1, and Targetscan6.2) to build novel, comparative platforms of binding sites for the promoter (4 prediction datasets), cds (5 prediction datasets), 5′-(5 prediction datasets), and 3′-UTR (13 prediction datasets) regions. Information on the miRNA-target interactions of 2035 disease ontologies (DO), 6727 human phenotype ontologies (HPO), and 4980 OMIM disorders is available. This information provides possible interactions between microRNAs and genes associated with the 597 KEGG, 456 Panther, and 522 Wiki pathways.

## 5. Conclusions

In conclusion, a history of preterm delivery (preterm prelabor rupture of membranes and/or spontaneous preterm birth) is second only to a history of GDM for association with more severe postpartal alterations in microRNA expression profiles when compared with a history of other pregnancy-related complications (gestational hypertension, preeclampsia, and fetal growth restriction). The earlier the preterm delivery occurs, and the lower the birth weight of the newborn, the higher the postpartal microRNA expression levels may be in the peripheral white blood cells. Similarly, imminent preterm delivery is associated with much higher postpartal microRNA expression levels than postponed preterm delivery with use of tocolytics and antenatal corticosteroids. In addition, mothers with serum CRP levels above 20 mg/L in preterm labour had more altered postpartal microRNA expression profiles than mothers with normal serum CRP levels in preterm labour. These findings may contribute to understanding the increased cardiovascular risk in mothers with anamnesis of preterm delivery and to explaining the highest maternal cardiovascular risk in mothers with anamnesis of extremely preterm and very preterm delivery. Nevertheless, it is clear that consecutive, large-scale studies to verify these initial findings are needed.

## 6. Patents

National patent granted: Industrial Property Office, Czech Republic (Patent n. 308178).

International patent filed: Industrial Property Office, Czech Republic (PCT/CZ2019/050051).

## Figures and Tables

**Table 1:** Characteristics of cases and controls.

	NP (*n* = 89)	PPROM (*n* = 58)	PTB (*n* = 55)	*p*-Value ^1^	*p*-Value ^2^
At Follow-Up					
Age (years)	38 (29–50)	39 (26–52)	38 (25–48)	0.512	0.998
Time since index pregnancy (years)	5 (3–11)	6 (3–9)	6 (3–10)	0.090	0.092
Height (cm)	167.0 (153–181)	166.0 (146–180)	167.0 (154.5–182)	0.244	0.494
Weight (kg)	62.7 (46–109)	64.65 (43.5–110)	62.9 (46.9–104)	0.367	0.930
Body mass index (BMI) (kg/m^2^)	22.23 (17.7–39.08)	23.82 (17.36–39.92)	21.97 (18.25–36.85)	0.082	0.918
Systolic blood pressure (BP) (mmHg)	112 (87–138)	112.5 (96–189)	113 (96–144)	0.869	0.912
Diastolic BP (mmHg)	71 (55–91)	74.5 (62–112)	72 (61–104)	0.058	0.441
Hypertension on treatment	1 (1.12%)	0	1 (1.82%)	-	0.082
Diabetes mellitus	1 (1.12%)	1 (1.72%)	0	0.061	-
Dyslipidaemia	0	0	1 (1.82%)	-	-
Inborn kidney disease	0	0	1 (1.82%)	-	-
Birth defects of the heart Ventricular septal defect Foramen ovale apertum	0	1 (1.72%) 1 (1.72%)	1 (1.82%) 1 (1.82%)	-	-
Heart arrhythmia	0	2	0	-	-
Rheumatoid arthritis	0	1 (1.72%)	1 (1.82%)	-	-
Systemic lupus erythematosus nephrotic syndrome	0	1 (1.72%) 1 (1.72%)	0	-	-
Trombophilic gene mutations	1 (1.12%)	6 (10.34%)	8 (14.55%)	**0.010**	**0.001**
Deep venous thrombosis	1 (1.12%)	1 (1.72%)	1 (1.82%)	0.061	0.082
Pulmonary embolism	0	1 (1.72%)	0	-	-
Cerebrovascular accidents Recurrent cerebrovascular accidents	0	1 (1.72%) 1 (1.72%)	0	-	-
Immune thrombocytopenia	0	1 (1.72%)	0	-	-
Antiphospholipid syndrome	0	1 (1.72%)	0	-	-
**During gestation**					
Maternal age at delivery (years)	32 (25–43)	32 (22–44)	33 (20–40)	0.794	0.396
GA at delivery (weeks)	39.86 (37.71–41.86)	33.07 (24.71–35.86)	31.43 (24–36.43)	**<0.001**	**<0.001**
Mode of delivery					
Vaginal	82 (92.13%)	27 (46.55%)	38 (69.09%)	**<0.001**	**<0.001**
Caesarean section (CS)	7 (7.87%)	31 (53.45%)	17 (30.91%)		
Fetal birth weight (g)	3410 (2530–4450)	1980 (600–2710)	1580 (542–2900)	**<0.001**	**<0.001**
Fetal sex					
Boy	47 (52.81%)	25 (43.10%)	37 (67.27%)	0.250	0.087
Girl	42 (47.19%)	33 (56.90%)	18 (32.73%)		
Primiparity at index pregnancy					
Yes	43 (48.31%)	38 (65.52%)	33 (60.00%)	0.040	0.172
No	46 (51.69%)	20 (34.48%)	22 (40.00%)		
Birth order of index pregnancy					
1st	35 (39.32%)	27 (46.55%)	21 (38.18%)	0.478	0.329
2nd	33 (37.08%)	17 (29.31%)	17 (30.91%)		
3rd	16 (17.98%)	8 (13.79%)	9 (16.36%)		
4th+	5 (5.62%)	6 (10.34%)	8 (14.55%)		
Total number of pregnancies per patient					
1	8 (8.99%)	11 (18.97%)	6 (10.91%)	0.188	0.592
2	45 (50.56%)	24 (41.38%)	23 (41.82%)		
3+	36 (40.45%)	23 (39.65%)	26 (47.27%)		
Infertility treatment					
Yes	4 (4.49%)	9 (15.52%)	8 (14.55%)	**0.021**	**0.034**
No	85 (95.51%)	49 (84.48%)	47 (85.45%)		
Administration of corticosteroids					
Yes	-	45 (77.59%)	39 (70.91%)		
No	-	13 (22.41%)	16 (29.09%)		
Administration of antibiotics					
Yes	-	54 (93.10%)	36 (65.45%)		
No	-	4 (6.90%)	19 (34.55%)		
Tocolytic therapy					
Yes	-	30 (51.72%)	36 (65.45%)		
No	-	28 (48.28%)	19 (34.55%)		
CRP levels > 20 mg/L	-	3 (5.17%)	9 (16.36%)		
WBC count > 16.9 × 10^9^/L	-	6 (10.34%)	10 (18.18%)		
Apgar score <7, 5 min	0	4 (6.90%)	2 (3.64%)		
Apgar score <7, 10 min	0	4 (6.90%)	1 (1.82%)		
Umbilical blood pH	7.3 (7.29–7.3)	7.3 (6.9–7.4)	7.3 (6.8–7.5)	0.291	0.273

Data are presented as the median (range) for continuous variables and as the number (percentage) for categorical variables. Statistically significant results are marked in bold. Continuous variables were compared using the Kruskal-Wallis test. Categorical variables were compared using the chi-square test. *p*-value ^1^: the comparison between normal pregnancies and PPROM. *p*-value ^2^: the comparison between normal pregnancies and PTB. NP, normal pregnancies. PTB, spontaneous preterm birth. PPROM, preterm prelabor rupture of membranes. BMI, body mass index. BP, blood pressure. CRP, C-reactive protein. CS, caesarean section. GA, gestational age. WBC, white blood cells. “+” means that birth order of the index pregnancy is 4th or more.

**Table 2:** MicroRNA gene expression in women with a history of preterm prelabor rupture of membranes (PPROM) and preterm birth (PTB): Pilot and validation experimental data analyses.

	Pilot Study PPROM (*n* = 10) vs. NP (*n* = 10) PTB (*n* = 10) vs. NP (*n* = 10)	Validation Study PPROM (*n* = 10) vs. NP (*n* = 10) PTB (*n* = 10) vs. NP (*n* = 10)
*miR-1-3p*	1.124 ± 1.870 vs. 0.041 ± 0.022, ***p* < 0.001** 1.234 ± 1.170 vs. 0.041 ± 0.022, ***p* < 0.001**	1.690 ± 1.337 vs. 0.047 ± 0.061, ***p* < 0.001** 0.631 ± 0.594 vs. 0.047 ± 0.061, ***p* < 0.001**
*miR-16-5p*	1.755 ± 0.542 vs. 0.904 ± 0.317, ***p* = 0.001** 1.848 ± 0.736 vs. 0.904 ± 0.317, ***p* = 0.003**	2.294 ± 0.933 vs. 0.792 ± 0.648, ***p* < 0.001** 1.538 ± 0.531 vs. 0.792 ± 0.648, ***p* = 0.010**
*miR-17-5p*	2.268 ± 0.788 vs. 1.115 ± 0.491, ***p* = 0.002** 2.168 ± 0.048 vs. 1.115 ± 0.491, ***p* = 0.003**	2.778 ± 1.264 vs. 0.485 ± 0.337, ***p* < 0.001** 1.871 ± 0.777 vs. 0.485 ± 0.337, ***p* < 0.001**
*miR-20a-5p*	1.998 ± 1.688 vs. 0.817 ± 0.364, ***p* = 0.008** 7.248 ± 8.752 vs. 0.817 ± 0.364, *****p* = 0.005****	3.737 ± 2.586 vs. 0.985 ± 1.145, ***p* = 0.003** 1.769 ± 0.979 vs. 0.985 ± 1.145, ***p* = 0.023**
*miR-20b-5p*	2.726 ± 1.824 vs. 0.806 ± 0.525, ***p* = 0.001** 3.167 ± 2.111 vs. 0.806 ± 0.525, ***p*<** **0.001**	3.163 ± 1.431 vs. 0.660 ± 0.548, ***p* < 0.001** 1.734 ± 0.700 vs. 0.660 ± 0.548, ***p* = 0.004**
*miR-21-5p*	0.569 ± 0.235 vs. 0.188 ± 0.079, ***p* < 0.001** 0.489 ± 0.328 vs. 0.188 ± 0.079, ***p* = 0.015**	0.493 ± 0.248 vs. 0.092 ± 0.091, ***p* < 0.001** 0.468 ± 0.121 vs. 0.092 ± 0.091, ***p* < 0.001**
*miR-23a-3p*	0.350 ± 0.241 vs. 0.119 ± 0.055, ***p* = 0.001** 0.420 ± 0.267 vs. 0.119 ± 0.055, ***p*<** **0.001**	0.441 ± 0.285 vs. 0.236 ± 0.175, ***p* = 0.049** 0.420 ± 0.331 vs. 0.236 ± 0.175, ***p* = 0.034**
*miR-24-3p*	0.394 ± 0.168 vs. 0.242 ± 0.129, ***p* = 0.034** 0.350 ± 0.169 vs. 0.242 ± 0.129, *p* = 0.058	0.448 ± 0.190 vs. 0.183 ± 0.205, ***p* = 0.001** 0.259 ± 0.087 vs. 0.183 ± 0.205, *p* = 0.082
*miR-26a-5p*	0.893 ± 0.448 vs. 0.303 ± 0.146, ***p* < 0.001** 0.706 ± 0.297 vs. 0.303 ± 0.146, ***p* = 0.003**	1.250 ± 0.492 vs. 0.200 ± 0.141, ***p* < 0.001** 0.815 ± 0.368 vs. 0.200 ± 0.141, ***p* < 0.001**
*miR-29a-3p*	0.571 ± 0.270 vs. 0.111 ± 0.047, ***p* < 0.001** 0.468 ± 0.190 vs. 0.111 ± 0.047, ***p* < 0.001**	0.701 ± 0.519 vs. 0.173 ± 0.161, ***p* = 0.001** 0.541 ± 0.128 vs. 0.173 ± 0.161, ***p* < 0.001**
*miR-92a-3p*	3.189 ± 1.214 vs. 2.169 ± 1.276, ***p* = 0.003** 2.718 ± 1.012 vs. 2.169 ± 1.276, ***p* = 0.041**	2.806 ± 2.086 vs. 1.981 ± 1.960, *p* = 0.112 1.923 ± 0.774 vs. 1.981 ± 1.960, *p* = 0.257
*miR-100-5p*	0.005 ± 0.004 vs. 0.001 ± 0.001, ***p* = 0.001** 0.003 ± 0.002 vs. 0.001 ± 0.001, ***p* < 0.001**	0.005 ± 0.003 vs. 0.002 ± 0.002, ***p* = 0.015** 0.004 ± 0.003 vs. 0.002 ± 0.002, ***p* = 0.023**
*miR-103a-3p*	2.523 ± 1.593 vs. 0.951 ± 0.439, ***p* < 0.001** 2.381 ± 0.973 vs. 0.951 ± 0.439, ***p* < 0.001**	3.175 ± 1.802 vs. 0.637 ± 0.636, ***p* < 0.001** 2.144 ± 0.942 vs. 0.637 ± 0.636, ***p* = 0.002**
*miR-125b-5p*	0.008 ± 0.007 vs. 0.003 ± 0.002, ***p* = 0.049** 0.006 ± 0.004 vs. 0.003 ± 0.002, ***p* = 0.015**	0.008 ± 0.005 vs. 0.001 ± 0.002, ***p* = 0.005** 0.006 ± 0.002 vs. 0.001 ± 0.002, ***p* = 0.005**
*miR-126-3p*	0.531 ± 0.346 vs. 0.158 ± 0.072, ***p* < 0.001** 0.350 ± 0.181 vs. 0.158 ± 0.072, ***p* = 0.005**	0.685 ± 0.387 vs. 0.130 ± 0.139, ***p* < 0.001** 0.446 ± 0.204 vs. 0.130 ± 0.139, ***p* = 0.001**
*miR-130b-3p*	1.024 ± 0.472 vs. 0.302 ± 0.171, ***p* < 0.001** 1.794 ± 1.331 vs. 0.302 ± 0.171, ***p* < 0.001**	1.339 ± 0.803 vs. 0.321 ± 0.332, ***p* = 0.002** 0.903 ± 0.210 vs. 0.321 ± 0.332, ***p* = 0.002**
*miR-133a-3p*	0.400 ± 0.616 vs. 0.125 ± 0.078, ***p* = 0.049** 0.328 ± 0.257 vs. 0.125 ± 0.078, *p* = 0.069	0.536 ± 0.513 vs. 0.035 ± 0.040, ***p* < 0.001** 0.399 ± 0.479 vs. 0.035 ± 0.040, ***p* < 0.001**
*miR-143-3p*	0.078 ± 0.056 vs. 0.010 ± 0.005, ***p* < 0.001** 0.057 ± 0.032 vs. 0.010 ± 0.005, ***p* < 0.001**	0.085 ± 0.055 vs. 0.015 ± 0.023, ***p* = 0.001** 0.073 ± 0.035 vs. 0.015 ± 0.023, ***p* < 0.001**
*miR-145-5p*	0.208 ± 0.180 vs. 0.120 ± 0.054, ***p* = 0.008** 0.160 ± 0.033 vs. 0.120 ± 0.054, ***p* = 0.019**	0.223 ± 0.136 vs. 0.055 ± 0.056, ***p* < 0.001** 0.164 ± 0.087 vs. 0.055 ± 0.056, ***p* = 0.001**
*miR-146a-5p*	1.844 ± 0.921 vs. 0.607 ± 0.281, ***p* < 0.001** 2.277 ± 1.526 vs. 0.607 ± 0.281, ***p* = 0.001**	2.761 ± 1.712 vs. 2.463 ± 3.856, ***p* = 0.034** 2.672 ± 1.040 vs. 2.463 ± 3.856, ***p* = 0.023**
*miR-155-5p*	0.839 ± 0.386 vs. 0.579 ± 0.162, *p* = 0.879 1.211 ± 1.000 vs. 0.579 ± 0.162, *p* = 0.879	2.234 ± 3.722 vs. 1.107 ± 0.414, *p* = 0.289 1.016 ± 0.582 vs. 1.107 ± 0.414, *p* = 0.821
*miR-181a-5p*	0.465 ± 0.325 vs. 0.159 ± 0.083, ***p* < 0.001** 0.525 ± 0.270 vs. 0.159 ± 0.083, ***p* < 0.001**	0.499 ± 0.225 vs. 0.141 ± 0.195, ***p* = 0.001** 0.379 ± 0.167 vs. 0.141 ± 0.195, ***p* = 0.002**
*miR-195-5p*	0.277 ± 0.318 vs. 0.022 ± 0.019, ***p* < 0.001** 0.397 ± 0.369 vs. 0.022 ± 0.019, ***p* < 0.001**	0.596 ± 0.600 vs. 0.026 ± 0.040, ***p* < 0.001** 0.164 ± 0.160 vs. 0.026 ± 0.040, ***p* = 0.003**
*miR-199a-5p*	0.132 ± 0.145 vs. 0.018 ± 0.008, ***p* < 0.001** 0.096 ± 0.071 vs. 0.018 ± 0.008, ***p* < 0.001**	0.306 ± 0.347 vs. 0.022 ± 0.040, ***p* < 0.001** 0.182 ± 0.347 vs. 0.022 ± 0.040, ***p* < 0.001**
*miR-210-3p*	0.136 ± 0.077 vs. 0.193 ± 0.129, *p* = 0.406 0.116 ± 0.077 vs. 0.193 ± 0.129, *p* = 0.326	0.118 ± 0.079 vs. 0.165 ± 0.241, *p* = 0.364 0.109 ± 0.046 vs. 0.165 ± 0.241, *p* = 0.198
*miR-221-3p*	0.932 ± 0.666 vs. 0.285 ± 0.185, ***p* = 0.002** 0.694 ± 0.374 vs. 0.285 ± 0.185, ***p* = 0.006**	1.096 ± 0.601 vs. 0.280 ± 0.221, ***p* < 0.001** 0.641 ± 0.250 vs. 0.280 ± 0.221, ***p* = 0.003**
*miR-342-3p*	3.563 ± 1.393 vs. 2.899 ± 1.415, *p* = 0.173 3.191 ± 2.279 vs. 2.899 ± 1.415, *p* = 0.151	3.507 ± 1.378 vs. 1.866 ± 1.591, ***p* = 0.019** 2.551 ± 1.103 vs. 1.866 ± 1.591, ***p* = 0.041**
*miR-499a-5p*	0.983 ± 0.624 vs. 0.039 ± 0.028, ***p* < 0.001** 0.554 ± 0.352 vs. 0.039 ± 0.028, ***p* < 0.001**	0.847 ± 0.679 vs. 0.071 ± 0.145, ***p* < 0.001** 0.794 ± 0.373 vs. 0.071 ± 0.145, ***p* < 0.001**
*miR-574-3p*	0.256 ± 0.118 vs. 0.099 ± 0.054, ***p* = 0.002** 0.257 ± 0.128 vs. 0.099 ± 0.054, ***p* < 0.001**	0.323 ± 0.168 vs. 0.099 ± 0.085, ***p* = 0.004** 0.175 ± 0.064 vs. 0.099 ± 0.085, ***p* = 0.019**

MicroRNA gene expression was compared between individual groups using the Mann-Whitney test. Statistically significant results are marked in bold. Mean ± SD values of the relative fold gene expression of samples (2^–∆∆Ct^) are presented. NP, normal pregnancies. PTB, spontaneous preterm birth. PPROM, preterm prelabor rupture of membranes.

**Table 3:** Benjamini-Hochberg correction for multiple comparisons. (**a**) Comparison of postpartal microRNA gene expression between women with a history of preterm and term deliveries (normal pregnancies, PPROM, PTB). (**b**) Comparison of postpartal microRNA gene expression between women with a history of preterm and term deliveries, based on gestational age (normal pregnancies, extremely preterm birth, very preterm birth, and moderate to late preterm birth).

**(a)**				
**K**	**i**	**Alpha = 0.05**	**Alpha = 0.01**	**Alpha = 0.001**
3		0.05	0.01	0.001
	1	0.017	0.003	0.000
	2	0.033	0.007	0.001
	3	0.050	0.010	0.001
**(b)**				
**K**	**i**	**Alpha = 0.05**	**Alpha = 0.01**	**Alpha = 0.001**
6		0.05	0.01	0.001
	1	0.008	0.002	0.000
	2	0.017	0.003	0.000
	3	0.025	0.005	0.001
	4	0.033	0.007	0.001
	5	0.042	0.008	0.001
	6	0.050	0.010	0.001

**Table 4:** Postpartal increase of expression of diabetes/cardiovascular/cerebrovascular disease-associated microRNAs in the peripheral white blood cells of mothers with descending gestational age at delivery.

microRNA	ρ	*p*-Value
miR-1-3p	–0.622	*p* < 0.001
miR-16-5p	–0.441	*p* < 0.001
miR-17-5p	–0.453	*p* < 0.001
miR-20a-5p	–0.409	*p* < 0.001
miR-20b-5p	–0.482	*p* < 0.001
miR-21-5p	–0.447	*p* < 0.001
miR-23a-3p	–0.383	*p* < 0.001
miR-24-3p	–0.249	*p* < 0.001
miR-26a-5p	–0.479	*p* < 0.001
miR-29a-3p	–0.489	*p* < 0.001
miR-92a-3p	–0.162	*p* = 0.021
miR-100-5p	–0.457	*p* < 0.001
miR-103a-3p	–0.526	*p* < 0.001
miR-125b-5p	–0.375	*p* < 0.001
miR-126-3p	–0.502	*p* < 0.001
miR-130b-3p	–0.472	*p* < 0.001
miR-133a-3p	–0.439	*p* < 0.001
miR-143-3p	–0.507	*p* < 0.001
miR-145-5p	–0.392	*p* < 0.001
miR-146a-5p	–0.428	*p* < 0.001
miR-181a-5p	–0.557	*p* < 0.001
miR-195-5p	–0.487	*p* < 0.001
miR-199a-5p	–0.490	*p* <0.001
miR-221-3p	–0.427	*p* < 0.001
miR-342-3p	–0.149	*p* = 0.035
miR-499a-5p	–0.535	*p* < 0.001
miR-574-3p	–0.371	*p* < 0.001

ρ, Spearman’s rank correlation coefficient.

**Table 5:** Postpartal increase of the expression of diabetes/cardiovascular/cerebrovascular disease-associated microRNAs in the peripheral white blood cells of mothers with descending birth weight of the newborns.

microRNA	ρ	*p*-Value
miR-1-3p	–0.643	*p* < 0.001
miR-16-5p	–0.472	*p* < 0.001
miR-17-5p	–0.501	*p* < 0.001
miR-20a-5p	–0.434	*p* < 0.001
miR-20b-5p	–0.526	*p* < 0.001
miR-21-5p	–0.495	*p* < 0.001
miR-23a-3p	–0.455	*p* < 0.001
miR-24-3p	–0.266	*p* < 0.001
miR-26a-5p	–0.512	*p* < 0.001
miR-29a-3p	–0.507	*p* < 0.001
miR-92a-3p	–0.195	***p* = 0.005**
miR-100-5p	–0.509	*p* < 0.001
miR-103a-3p	–0.557	*p* < 0.001
miR-125b-5p	–0.420	*p* < 0.001
miR-126-3p	–0.530	*p* < 0.001
miR-130b-3p	–0.516	*p* < 0.001
miR-133a-3p	–0.432	*p* < 0.001
miR-143-3p	–0.544	*p* < 0.001
miR-145-5p	–0.425	*p* < 0.001
miR-146a-5p	–0.446	*p* < 0.001
miR-181a-5p	–0.583	*p* < 0.001
miR-195-5p	–0.497	*p* < 0.001
miR-199a-5p	–0.552	*p* < 0.001
miR-221-3p	–0.459	*p* < 0.001
miR-342-3p	–0.179	*p* = 0.011
miR-499a-5p	–0.587	*p* < 0.001
miR-574-3p	–0.411	*p* < 0.001

ρ, Spearman’s rank correlation coefficient.

**Table 6:** A list of predicted targets of microRNAs dysregulated in the peripheral white blood cells of women with a history of PPROM/PTB in relation to the apoptosis pathway, using the miRWalk2.0 database (data available in the KEGG, WIKI, and Panther pathways).

	Predicted Targets		
microRNA	KEGG Pathways	Wiki Pathways	Panther Pathways
miR-1	IKBKB, IL3, PIK3R5	CASP2, IKBKB, LTA	ATF2, BAG4, BCL2L10, IKBKB, MAP4K2, MAP4K3, PRKCE, PRKCG
miR-16-5p	BCL2, IKBKB, IRAK2, PRKAR1A, PRKAR2A	BCL2, IKBKB	BCL2, CRADD, IKBKB
miR-17-5p	ATM, CASP6, CASP7, CASP8, CASP10, CFLAR, CYCS, DFFA, EXOG, FASLG, IL1R1, IRAK1, IRAK4, MAP3K14, PIK3R2, PPP3CA, PRKAR2A, PRKX, TNFRSF10A, TNFRSF10D, XIAP	BNIP3L, CASP6, CASP7, CASP8, CASP10, CFLAR, CYCS, DFFA, FASLG, IRF1, TNFRSF1B, TNFRSF21, XIAP	ATF6, BAG1, CASP7, CASP8, CASP10, CFLAR, CREB1, CREM, CYCS, EIF2S1, FASLG, HSPA5, MAP3K14, MAPK9, PRKCQ, REL, TNFRSF10D, TNFRSF1B, XIAP
miR-20a-5p	CHP2, PPP3R1	BCL2L11, CASP2, MCL1, TNFRSF21, TP73	BCL2L11, HSPA6, HSPA8, MAP3K5, MCL1, PRKCA, TMBIM6
miR-20b-5p	ATM, CASP6, CASP7, CASP8, CASP10, CFLAR, CYCS, DFFA, EXOG, FASLG, IL1R1, IRAK1, IRAK4, MAP3K14, PIK3R2, PPP3CA, PRKAR2A, PRKX, TNFRSF10A, TNFRSF10D, XIAP	BNIP3L, CASP6, CASP7, CASP8, CASP10, CFLAR, CYCS, DFFA, FASLG, IRF1, TNFRSF1B, TNFRSF21	ATF6, BAG1, CASP7, CASP8, CASP10, CFLAR, CREB1, CREM, CYCS, EIF2S1, FASLG, HSPA5, MAP3K14, MAPK9, PRKCQ, REL, TNFRSF10D, TNFRSF1B, XIAP
miR-21-5p	APAF1, CFLAR, FASLG	APAF1, CFLAR, FASLG, MAP3K1	APAF1, CFLAR, DAXX, EIF2S1, FASLG, MAP2K3
miR-23a-3p	CFLAR, EXOG, IKBKB, PIK3CB, TNFRSF10, TNFSF10B	CFLAR, IGF1, IKBKB, MAP3K1, TNFRSF10, TNFSF10B	BIK, CFLAR, CREM, EIF2AK2, IKBKB, MAP3K5, PIK3CB, TNFRSF10, TNFSF10B
miR-24-3p	BCL2L1, EXOG, FASLG, IKBKB, IL1B, IRAK4, MYD88, PIK3CB, RIPK1	BBC3, BCL2L2, BCL2L11, BNIP3L, FASLG, IKBKB, MYC, NFKBIE, RIPK1, TRAF1, TRAF3	BCL2L1, BCL2L2, BCL2L11, EIF2AK2, FASLG, FOS, IKBKB, PIK3CB, PRKCA, PRKCH, RIPK1
miR-26a-5p	APAF1, BID, BIRC2, CASP6, DFFB, PPP3CB, PPP3CC	APAF1, BAK1, BID, BIRC2, CASP6, CRADD, DFFB, MDM2, PMAIP1	APAF1, ATF2, BAG4, BAK1, BID, BIRC2, CRADD, CREB1, EIF2AK2, FOS, HSPA8, PRKCD, PRKCQ, RELB
miR-29a-3p	CASP8, CYCS, IL1RAP, TNFRSF1A	BAK1, CASP8, CYCS, HRK, IGF1, MCL1, TNFRSF1A	BAK1, CASP8, CYCS, HSPA5, MCL1, TNFRSF1A
miR-100-5p	IRAK3, PPP3CA	-	RELB
miR-103a-3p	IL1RAP, IL3, PRKAR1A	BCL2L2, CASP2, CRADD, IRF1, IRF5, TNFRSF25	ATF6, BCL2L10, BCL2L2, BIK, CRADD, HSPA1B, LTB, MADD, MAP2K3, MAPK3, PRKCD
miR-125b-5p	AIFM1, CAPN1, CASP9, CSF2RB, EXOG, IKBKG	BAK1, CASP2, CASP9, IKBKG, IRF4, MCL1, PRF1	AIFM1, BAG4, BAK1, CASP9, MADD, MCL1, PRKRA, REL, TMBIM6
miR-126-3p	TNFRSF10B	TNFRSF10B	-
miR-130b-3p	CHUK, PIK3CA	CHUK, TNFRSF1B, TP73	CREB1, CHUK, PIK3CA, TNFRSF1B
miR-133a-3p	ENDOD1, IRAK3, MAP3K14, TNFRSF10B	BCL2L2, BNIP3L, TNFRSF10B	BCL2L2, MAP3K14, TNFRSF10B
miR-143-3p	APAF1, BIRC2, BIRC3, TNFRSF10B, TNFRSF10D	APAF1, BIRC2, BIRC3, TNFRSF10B	APAF1, BAG1, BIRC2, BIRC3, MAPK3, MAPK9, PRKCE, TNFRSF10D
miR-145-5p	AIFM1, PIK3R5, TNFRSF10B	TNFRSF10B, TNFRSF25	AIFM1, MAP4K2, TMBIM6, TNFRSF10B
miR-146a-5p	CASP7, CASP9, DFFA, IL3, IRAK1, IRAK4, PPP3R2, PRKACA	CASP2, CASP7, CASP9, DFFA, PMAIP1, PRF1	BAG1, CASP7, CASP9, HSPA1A, JDP2, PRKCE
miR-181a-5p	AKT3, ATM, CASP8, CSF2RB, ENDOD1, EXOG, IL1A, IL1R1, IL1RAP, PPP3R1, PRKAR2A, TRADD	CASP8, CRADD, IRF5, MDM2, PMAIP1, TP63, TRADD	AKT3, ATF2, CASP8, CRADD, DAXX, FOS, MAPK1, TRADD
miR-195-5p	BCL2, IKBKB, IRAK2, PRKAR1A, PRKAR2A	BCL2, CRADD, IKBKB	BCL2, CRADD, IKBKB
miR-199a-5p	IKBKG, PRKAR1A, PRKX, RELA, TNF, TRADD	BBC3, GZMB, IKBKG, RELA, TNF, TRADD, TRAF1	CREM, EIF2AK2, GZMB, MAPK9, PRKCA, RELA, RELB, TNF, TRADD
miR-221-3p	AKT3, APAF1, CASP10, IKBKG, IL1RAP, PIK3CD, PPP3R1, TNFSF10	APAF1, BNIP3L, CASP10, IKBKG, IRF4, MAPK10, MDM2, TNFSF10	AKT3, APAF1, ATF2, ATF4, CASP10, CREB1, MAPK10, PIK3CD, PRKCB, TNFSF10
miR-499a-5p	AKT2, IL1RAP, PIK3CD, PPP3CA, PRKAR1A	MDM2	AKT2, ATF2, HSPA8, PIK3CD, PRKCE, TMBIM6
miR-574-3p	-	TP63	LTB, MADD

**Table 7:** A list of predicted targets of microRNAs dysregulated in the peripheral white blood cells of women with a history of PPROM/PTB in relation to the inflammatory response pathway, using the miRWalk2.0 database (data available in WIKI pathways).

	Predicted Targets
microRNA	Wiki Pathways
miR-1	CD28, FN1, IL2RB
miR-16-5p	IL2RA
miR-17-5p	CD28, IL5, LAMC1, LAMC2, TNFRSF1B
miR-20a-5p	-
miR-20b-5p	CD28, IL5, LAMC1, LAMC2, TNFRSF1B
miR-21-5p	THBS3
miR-23a-3p	IFNG
miR-24-3p	CD28, CD86, FN1, IFNG, IL2RB, LAMC1
miR-26a-5p	COL1A2, IFNG
miR-29a-3p	COL1A2, IL2RA, LAMC1, TNFRSF1A
miR-100-5p	-
miR-103a-3p	CD40
miR-125b-5p	IL2RB
miR-126-3p	-
miR-130b-3p	LAMB2, TNFRSF1B
miR-133a-3p	CD28, FN1
miR-143-3p	CD28, CD40, IFNG, IL2RA
miR-145-5p	IL2RA
miR-146a-5p	CD80, CD86
miR-181a-5p	COL1A2, IL2, IL2RB, LAMC1
miR-195-5p	IL2RA
miR-199a-5p	IL4R
miR-221-3p	THBS1, VTN
miR-499a-5p	IL2RB, IL5RA
miR-574-3p	IL2RB

**Table 8:** A list of predicted targets of microRNAs dysregulated in the peripheral white blood cells of women with a history of PPROM/PTB in relation to the senescence and autophahy pathways, using the miRWalk2.0 database (data available in Wiki pathways).

	Predicted Targets
microRNA	Wiki Pathways
miR-1	ATG13, FN1, IL, LAMP2
miR-16-5p	BCL2, CREG1, HMGA1, LAMP2, MAP2K1, RAF1, SMAD4
miR-17-5p	ATG10, ATG12, CD44, CDKN1A, E2F1, IL6R, IRF1, LAMP2, RNASEL, RSL1D1, SERPINE1, SH3GLB1
miR-20a-5p	ATG14,ATG16L1, ATG5, ATG7, BECN1, BRAF, IGFBP7, IL8, RNASEL, RSL1D1, SH3GLB1, SQSTM1, ULK1
miR-20b-5p	ATG10, ATG12, CD44, CDKN1A, E2F1, IL6R, IRF1, LAMP2, RNASEL, RSL1D1, SERPINE1, SH3GLB1
miR-21-5p	MAP2K3
miR-23a-3p	AMBRA1, ATG13, BECN1, IFNG, IGF1, IL6R, IL8, MAPK14, PLAU
miR-24-3p	ATG13, CDKN1B, FN1, IFNG, IGFBP5, IL1B, IL6R, MAP1LC3A, MAP1LC3C, MMP14
miR-26a-5p	ATG13, COL10A1, HMGA1, IFNG, IL6, MDM2, PCNA, PTEN, RB1, ULK1
miR-29a-3p	IGF1, PTEN, RNASEL, SH3GLB1
miR-100-5p	MTOR
miR-103a-3p	ATG14, GABARAPL1, HMGA1, IL3, IRF1, IRF5, MAP2K3, SERPINB2, SMAD4
miR-125b-5p	AKT1S1, IGFBP3, RAF1
miR-126-3p	-
miR-130b-3p	AMBRA1, ATG14, CD44, IGFBP5, KMT2A, MAP2K1, MLST8, PTEN, RNASEL, SERPINE1
miR-133a-3p	ATG14, FN1, GABARAPL1, MAPK14, MMP14, RB1CC1, SLC39A1
miR-143-3p	ATG10, CD44, HMGA1, IFNG, IGFBP5, SERPINE1, SLC39A3
miR-145-5p	AMBRA1, CD44, HMGA1, IFNB1, MAP1LC3B, SLC39A2
miR-146a-5p	ATG12, IL3, KMT2A, RNASEL, SERPINB2, TNFSF15
miR-181a-5p	ATG10, ATG5, CDKN1B, CXCL1, IL1A, IRF5, MAPK1, MDM2, PTEN, ULK1
miR-195-5p	BCL2, CREG1, HMGA1, LAMP2, MAP2K1, RAF1, SMAD4
miR-199a-5p	CEBPB, IGFBP3, IL6, SLC39A3, UVRAG
miR-221-3p	-
miR-499a-5p	ATG3, MDM2, RB1
miR-574-3p	-

**Table 9:** A list of predicted targets of microRNAs dysregulated in the peripheral white blood cells of women with a history of PPROM/PTB in relation to thrombophilia, using the miRWalk2.0 database (data available in Disease Ontologies).

	Predicted Targets
microRNA	Disease Ontologies (DO)
miR-1	CPB2
miR-16-5p	-
miR-17-5p	MTHFR, SERPINE1
miR-20a-5p	-
miR-20b-5p	MTHFR, SERPINE1
miR-21-5p	-
miR-23a-3p	F8, F11
miR-24-3p	NOS3
miR-26a-5p	-
miR-29a-3p	-
miR-100-5p	-
miR-103a-3p	-
miR-125b-5p	-
miR-126-3p	-
miR-130b-3p	SERPINE1
miR-133a-3p	CPB2
miR-143-3p	CPB2, SERPINE1
miR-145-5p	-
miR-146a-5p	-
miR-181a-5p	F11, NOS3
miR-195-5p	-
miR-199a-5p	-
miR-221-3p	F11
miR-499a-5p	-
miR-574-3p	-

**Table 10:** A list of predicted targets of microRNAs dysregulated in the peripheral white blood cells of women with a history of PPROM/PTB in relation to infertility, using the miRWalk2.0 database (data available in Disease Ontologies and Human Phenotype Ontologies).

	Predicted Targets	
microRNA	Disease Ontologies (DO)	Human Phenotype Ontologies (HPO)
miR-1	BDNF, BOLL, CCL2, MUC1, PRSS21	-
miR-16-5p	CDC25A, KIF2C, PACRG, SGK1, TGIF2LY, VEGFA	DNAAF3, GBA2, RNF216
miR-17-5p	AHRR, ALOX15, BRCA1, CCL5, CD44, CD9, CGA, CREM, CX3CL1, HFE, HRH2, IL10, IL11, IL5, IL6R, LEP, MMP2, MTHFR, PCSK5, PRSS21, SERPINE1, SPACA1, UBE2B, VEGFA, XPC	CCDC40, CTNS, DNAI2, NANOS1, RNF216
miR-20a-5p	AHRR, BUB1, C5, ESR1, ITGA4, LIF, UBE2B	HSD17B3
miR-20b-5p	AHRR, ALOX15, BRCA1, CCL5, CD44, CD9, CGA, CREM, CX3CL1, HFE, HRH2, IL5, IL10, IL11, IL6R, LEP, MMP2, MTHFR, PCSK5, PRSS21, SERPINE1, SPACA1, UBE2B, VEGFA, XPC	CCDC40, CTNS, DNAI2, NANOS1, RNF216
miR-21-5p	CDC25A, OXTR, TLR4	-
miR-23a-3p	ARNT, CREM, FSHR, IL6R, MAS1, MBL2, MEFV, OAZ3, PUM2, SLC19A1, TLR4, TRO, XPA	HEATR2
miR-24-3p	ADM, CDC25A, IL1B, IL6R, NOS3, PAEP, PSG1, SCARB1, TFPI	CTNS, GBA2, HEATR2
miR-26a-5p	ADM, CCL2, ESR1, IL6, ITGA5, PACRG, SEMG2, SPATA16, SYCP3	CTNS, DYX1C1, PRLR, SPATA16
miR-29a-3p	DAZL, HRH1, LEP, OXTR, SGK1, VEGFA	-
miR-100-5p	CDC25A	-
miR-103a-3p	BDNF, CDC25A, LIPE, MLH3, TGIF2LY, TSSK6, WNT7A, XPC	-
miR-125b-5p	AURKC, LEP, LIF, MMP2, REC8, SCARB1	AURKC
miR-126-3p	-	-
miR-130b-3p	CATSPER1, CD44, EPPIN, FATE1, FMR1, LMNA, MBL2, MLH1, SERPINE1	CATSPER1
miR-133a-3p	FMR1, FOXL2, HRH1, SPO11, USP26, YBX2	GBA2
miR-143-3p	CD44, GALT, HRH2, ITGA6, MAS1, PACRG, PRDM9, PSG1, SERPINE1, TNP1	CCDC40, SNRPN
miR-145-5p	CD44, ESR2, GDNF, MUC1, WNT7A	C21orf59, NDN
miR-146a-5p	BRCA1, CCL5, FMR1, KIF2C, LHCGR, SPACA1	-
miR-181a-5p	AHR, AR, HLA-E, IL1A, KIF2C, LMNA, NOS3, PACRG, PRM1, TFPI	AR, CCDC40
miR-195-5p	CDC25A, KIF2C, PACRG, SGK1, TGIF2LY, VEGFA	DNAAF3, GBA2, RNF216
miR-199a-5p	CATSPER1, CREM, GANAB, GSTK1, IL6, LIF, LMNA, MMP9, REC8, SPACA1, TNF, TSSK6	CATSPER1, CCDC40, RNF216
miR-221-3p	NLRP3	-
miR-499a-5p	MLH1, REC8, SPACA1	H6PD
miR-574-3p	GAMT, LEP, MUC1	-

**Table 11:** A list of predicted targets of microRNAs dysregulated in the peripheral white blood cells of women with a history of PPROM/PTB in relation to susceptibility to hypertension, using the miRWalk2.0 database (data available in OMIM disorders).

	Predicted Targets
microRNA	OMIM disorders
miR-1	-
miR-16-5p	-
miR-17-5p	ECE1, PTGIS
miR-20a-5p	CYP3A5
miR-20b-5p	ECE1, PTGIS
miR-21-5p	-
miR-23a-3p	-
miR-24-3p	NOS3
miR-26a-5p	-
miR-29a-3p	NOS2
miR-100-5p	-
miR-103a-3p	-
miR-125b-5p	GNB3
miR-126-3p	-
miR-130b-3p	-
miR-133a-3p	-
miR-143-3p	GNB3
miR-145-5p	-
miR-146a-5p	-
miR-181a-5p	ATP1B1, NOS3
miR-195-5p	-
miR-199a-5p	PTGIS
miR-221-3p	-
miR-499a-5p	-
miR-574-3p	-

**Table 12:** A list of predicted targets of microRNAs dysregulated in the peripheral white blood cells of women with a history of PPROM/PTB in relation to susceptibility to obesity, using the miRWalk2.0 database (data available in OMIM disorders).

	Predicted Targets
microRNA	OMIM disorders
miR-1	-
miR-16-5p	-
miR-17-5p	-
miR-20a-5p	UCP3
miR-20b-5p	PPARGC1B
miR-21-5p	-
miR-23a-3p	-
miR-24-3p	PPARG
miR-26a-5p	PPARGC1B
miR-29a-3p	-
miR-100-5p	PPARGC1B
miR-103a-3p	ADRB2
miR-125b-5p	ENPP1, FFAR4
miR-126-3p	-
miR-130b-3p	PPARGC1B, SIM1, UCP3
miR-133a-3p	-
miR-143-3p	ADRB2, ENPP1
miR-145-5p	-
miR-146a-5p	-
miR-181a-5p	-
miR-195-5p	-
miR-199a-5p	-
miR-221-3p	-
miR-499a-5p	-
miR-574-3p	PPARGC1B

## Data Availability

The data presented in this study are available on request from the corresponding author. The data are not publicly available due to rights reserved by funding supporters.

## Supplementary Materials

Supplementary Materials can be found at https://www.mdpi.com/article/10.3390/ijms22084033/s1.

## Abbreviations

IL-6	Interleukin 6
HR	Hazard ratio
RR	Risk ratio
CRP	C-reactive protein
AUC	Area under the Curve
ROC	Receive Operating Characteristic
FPR	False Positive Rate
CI	Confidence Interval
LR+	Positive Likelihood Ratio
LR-	Negative Likelihood Ratio
NP	Normal Pregnancies
BP	Blood Pressure
SBP	Systolic Blood Pressure
DBP	Diastolic Blood Pressure
PPROM	Preterm prelabor rupture of membranes
PTB	Spontaneous preterm birth
CS	Caesarean section
AS	Apgar score
CVD	Cardiovascular disease
WBC	White blood cells
