# Supplementary material for: A History of Preterm Delivery Is Associated with Aberrant Postpartal MicroRNA Expression Profiles in Mothers with an Absence of Other Pregnancy-Related Complications
Source: Int J Mol Sci. 2021 Apr 14;22(8):4033. doi: 10.3390/ijms22084033 (PMC8070839; doi:10.3390/ijms22084033)
Supplement: Supplementary file 1 [file ijms-22-04033-s001.zip › Supplementary Material/Supplementary Figure S2.docx]

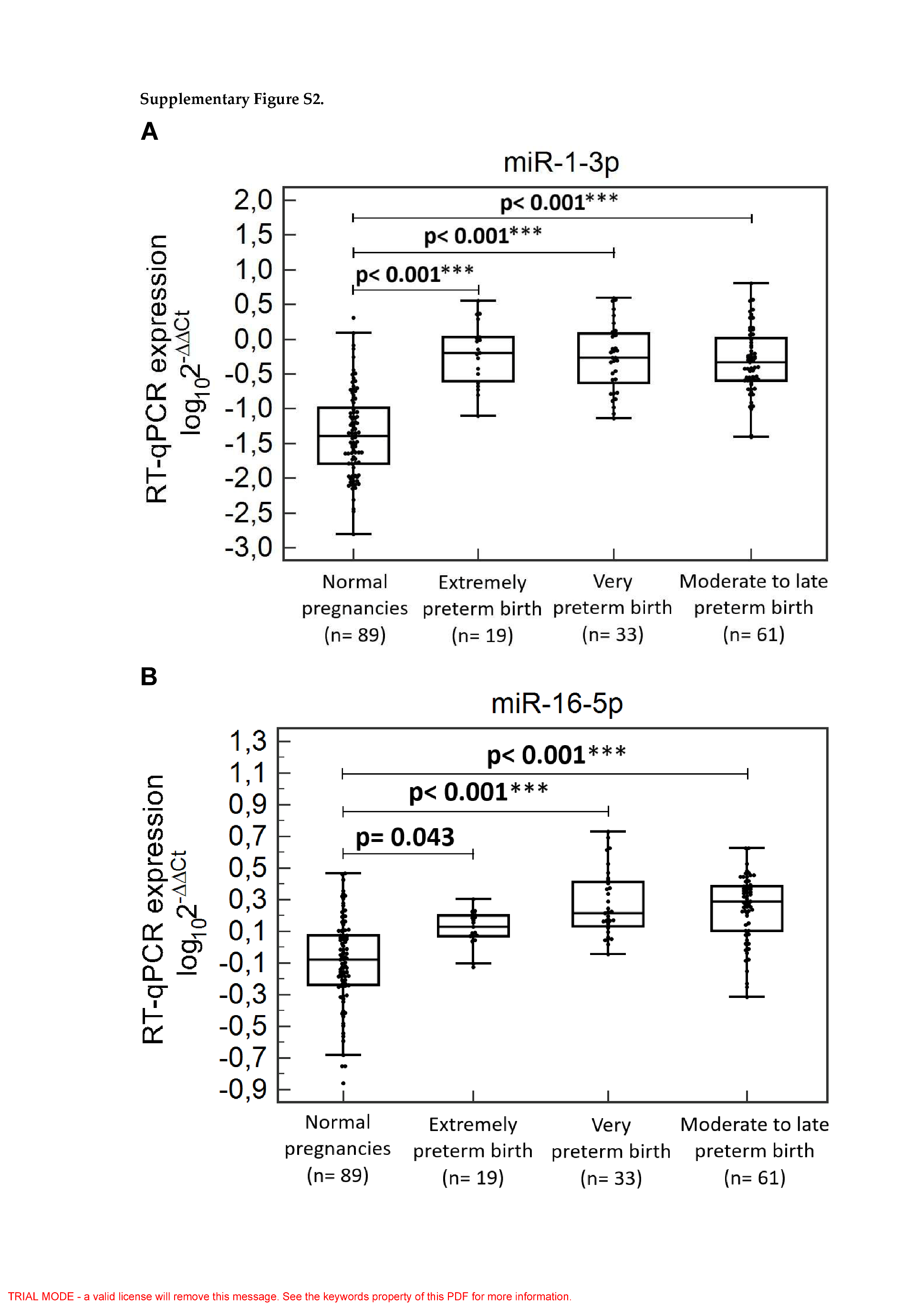


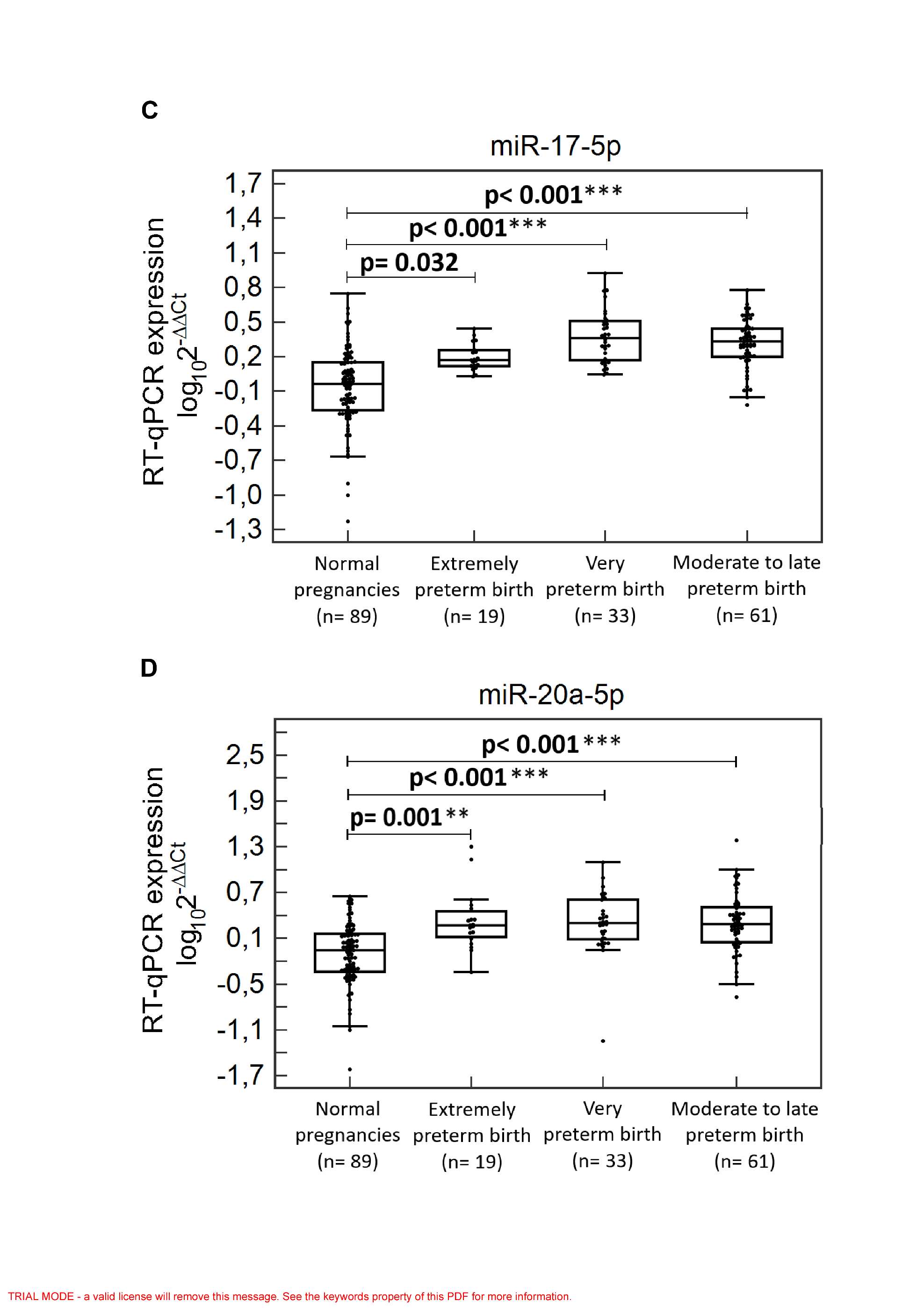


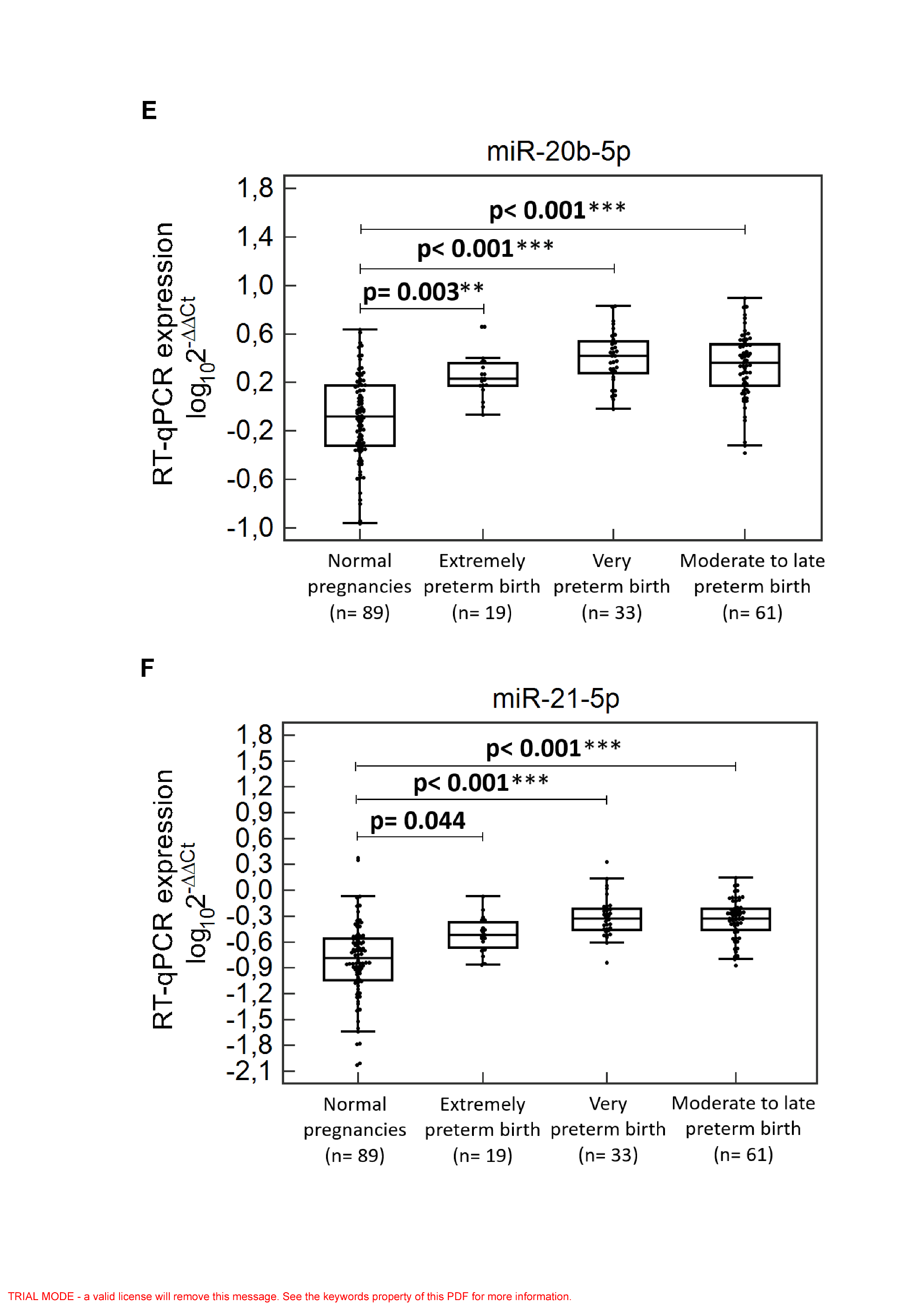


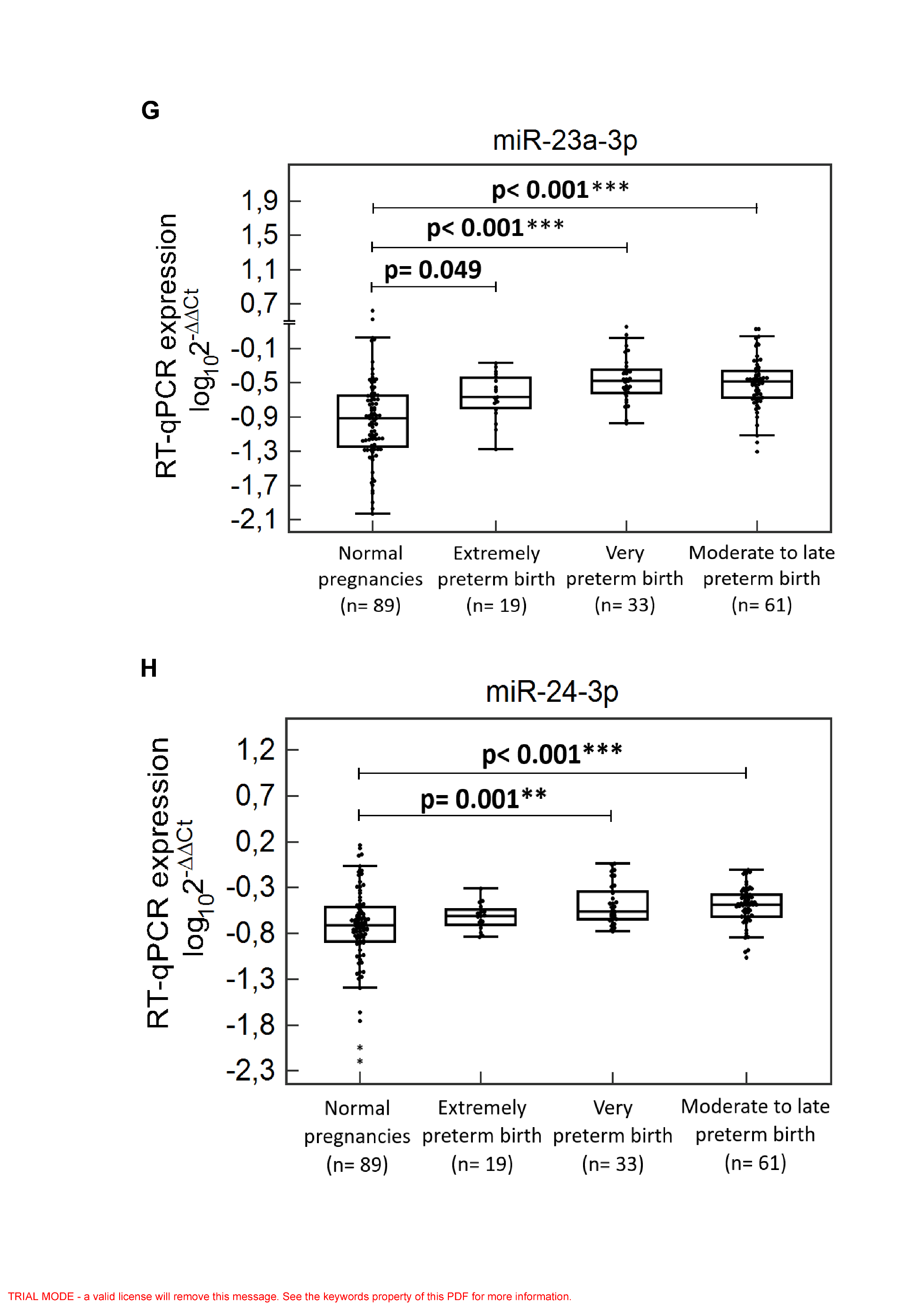


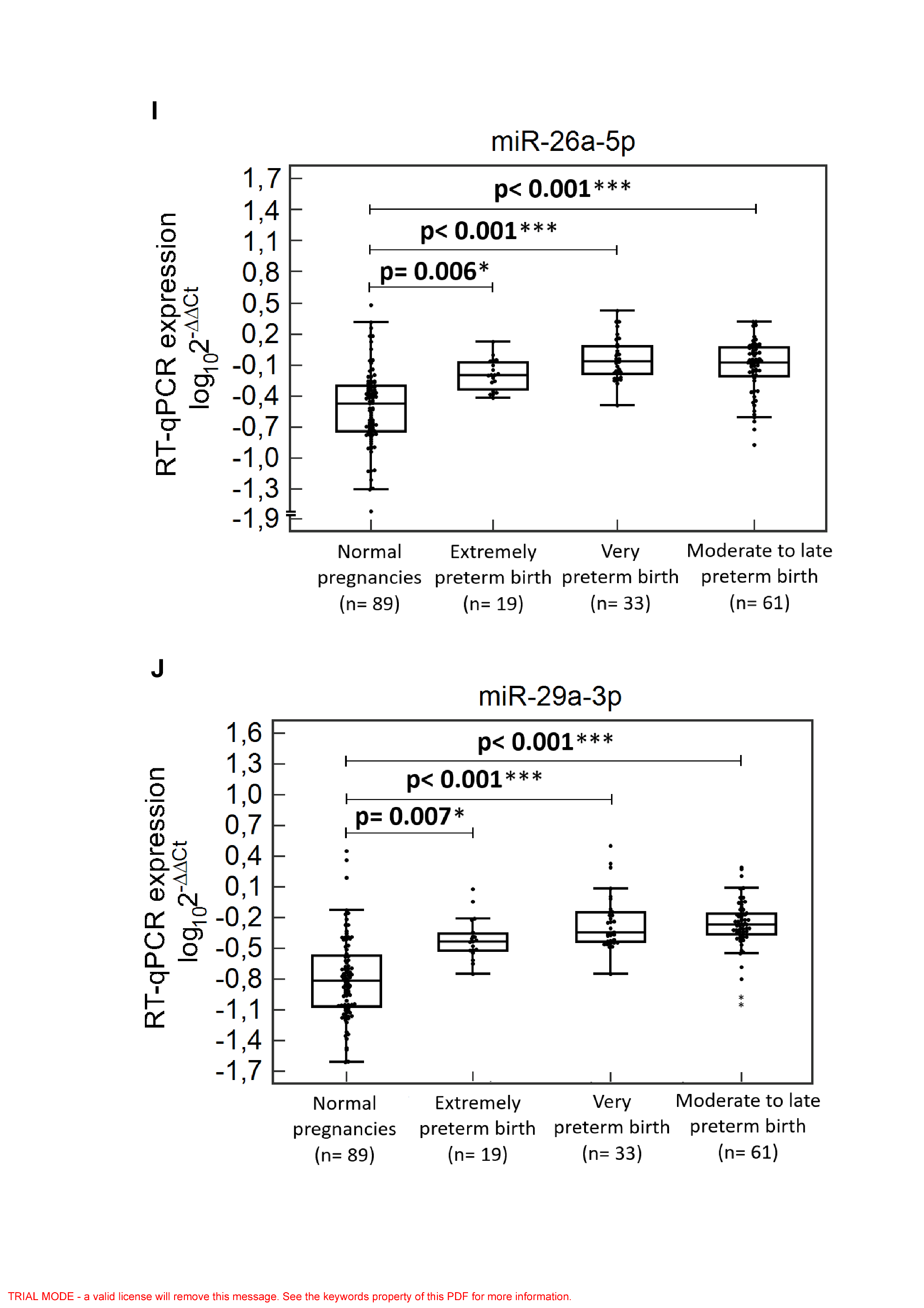


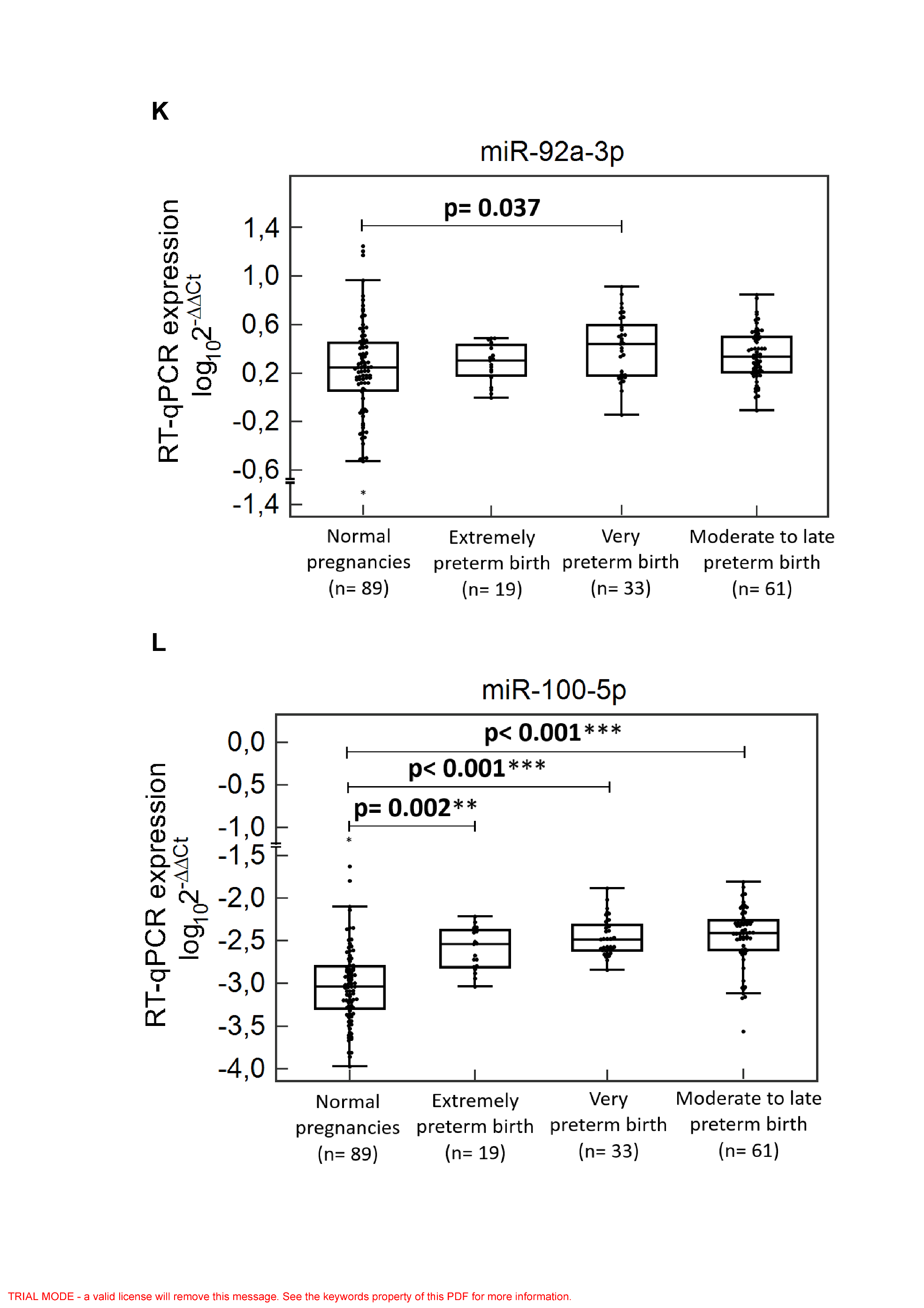


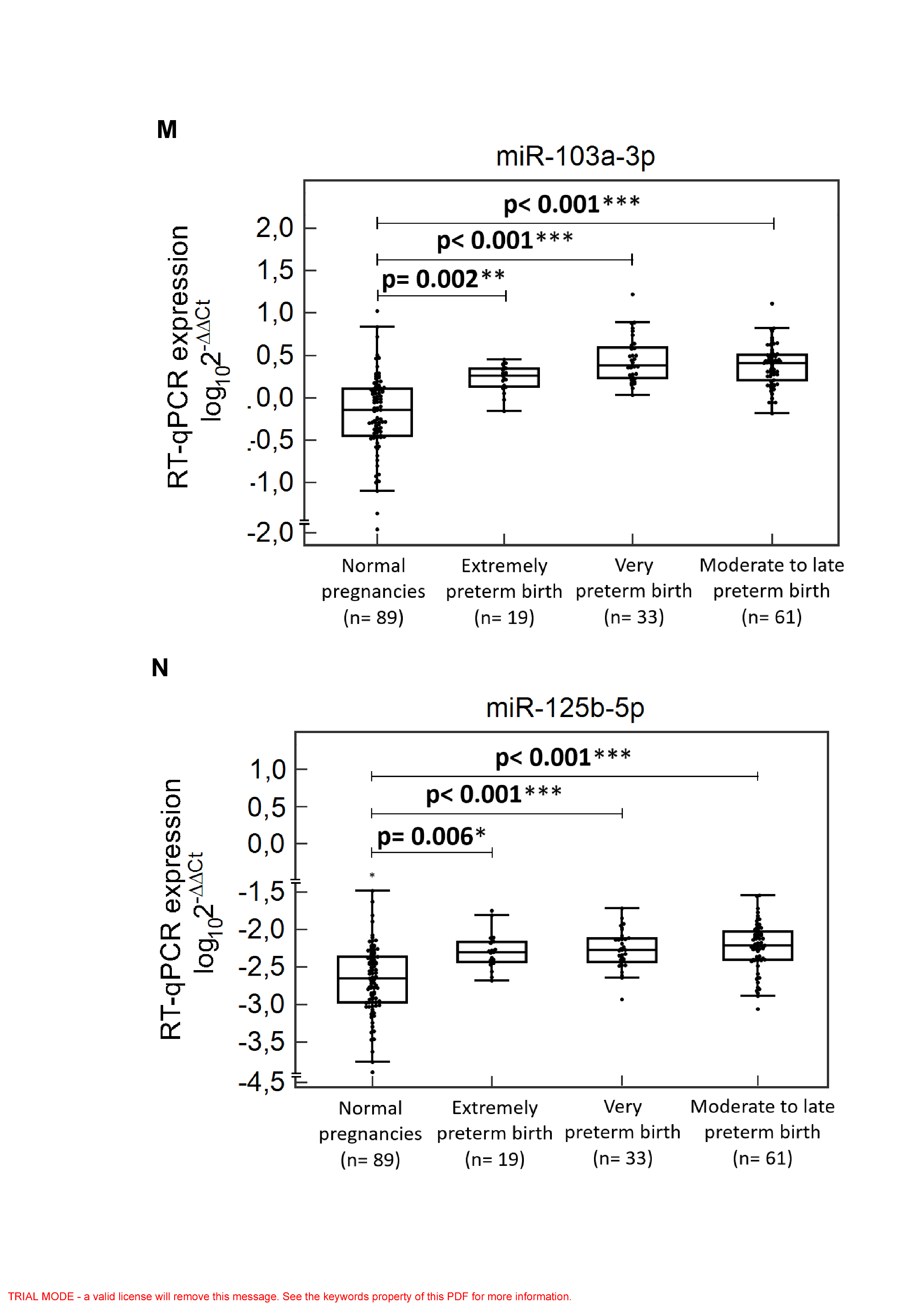


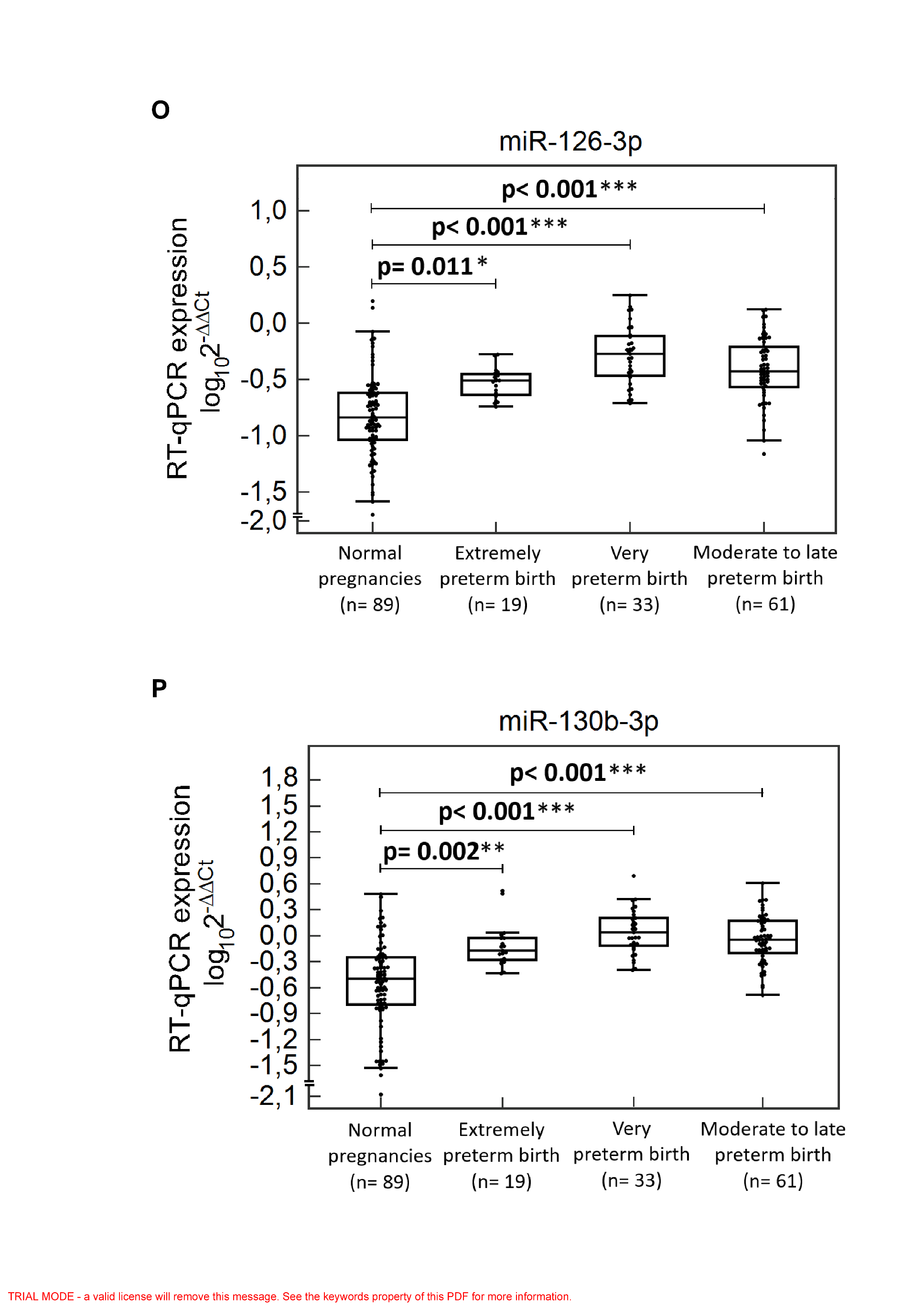


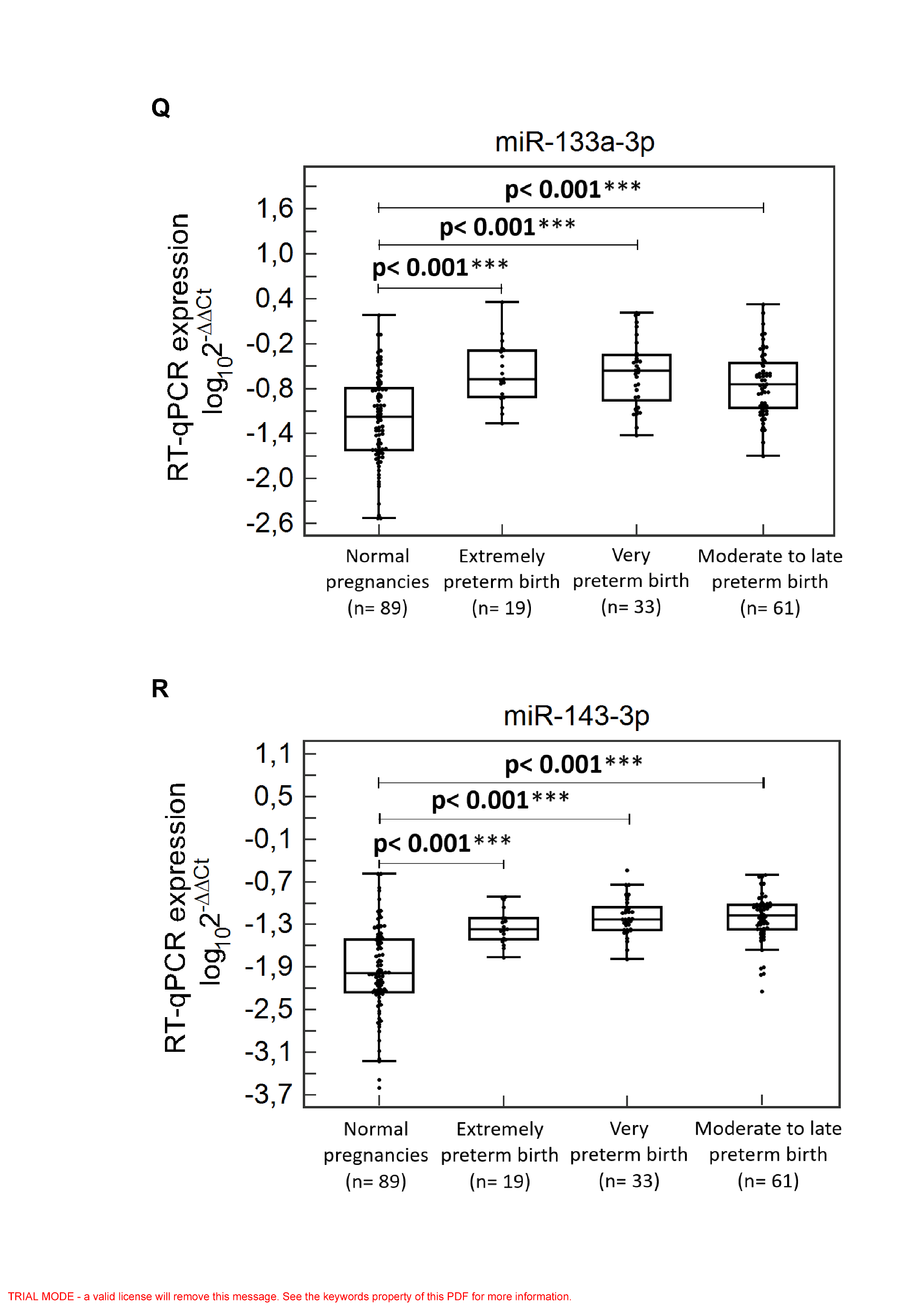


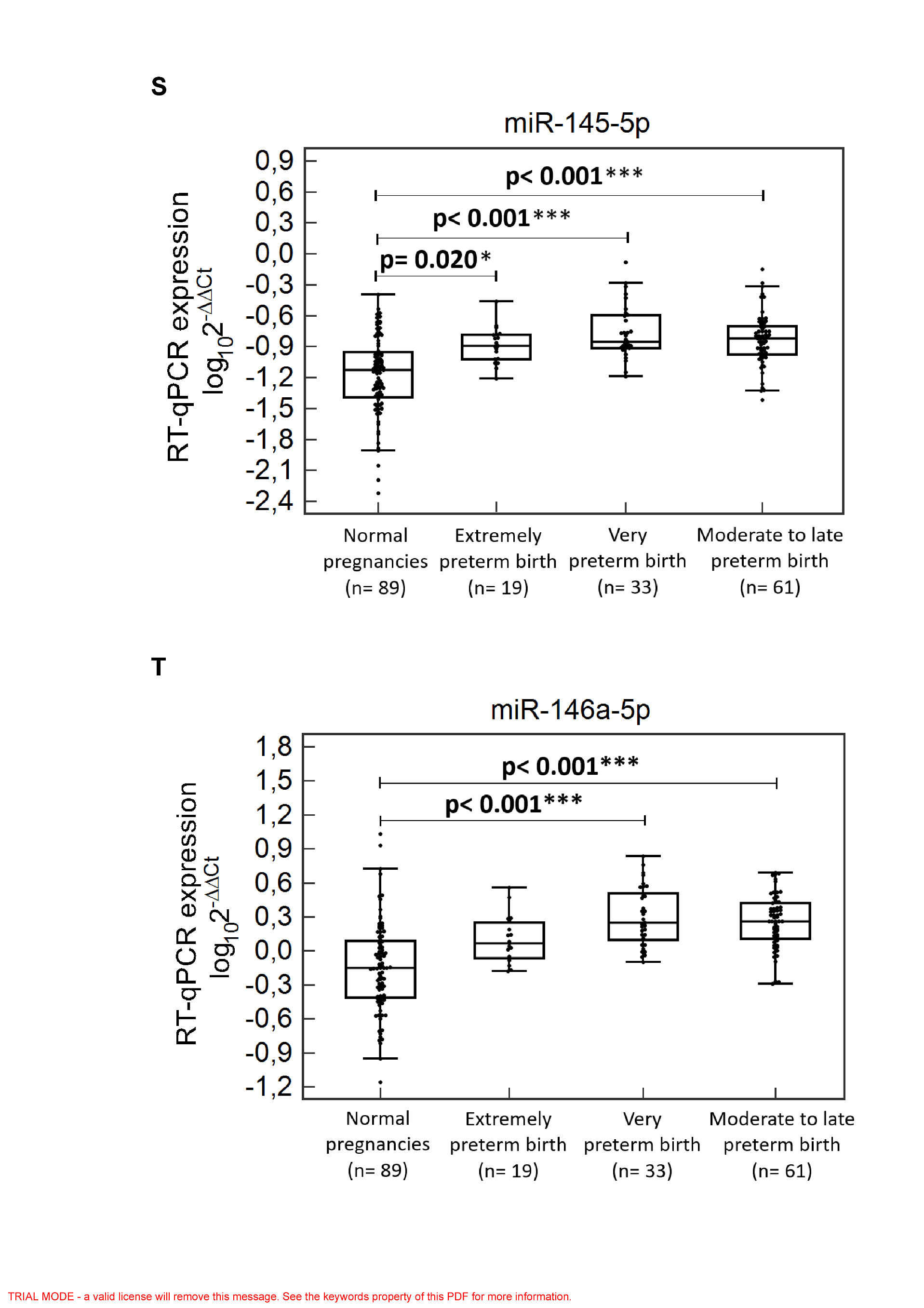


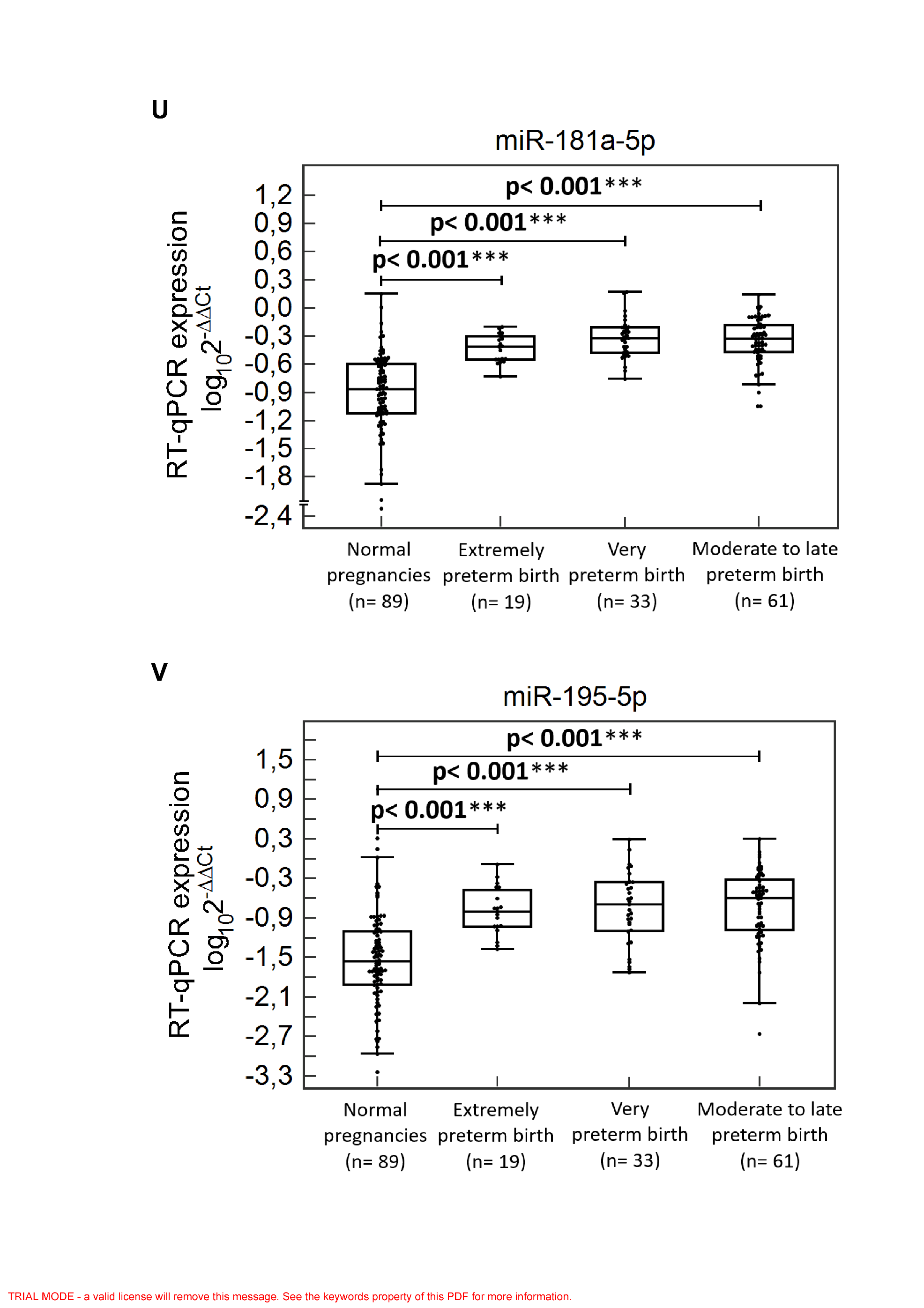


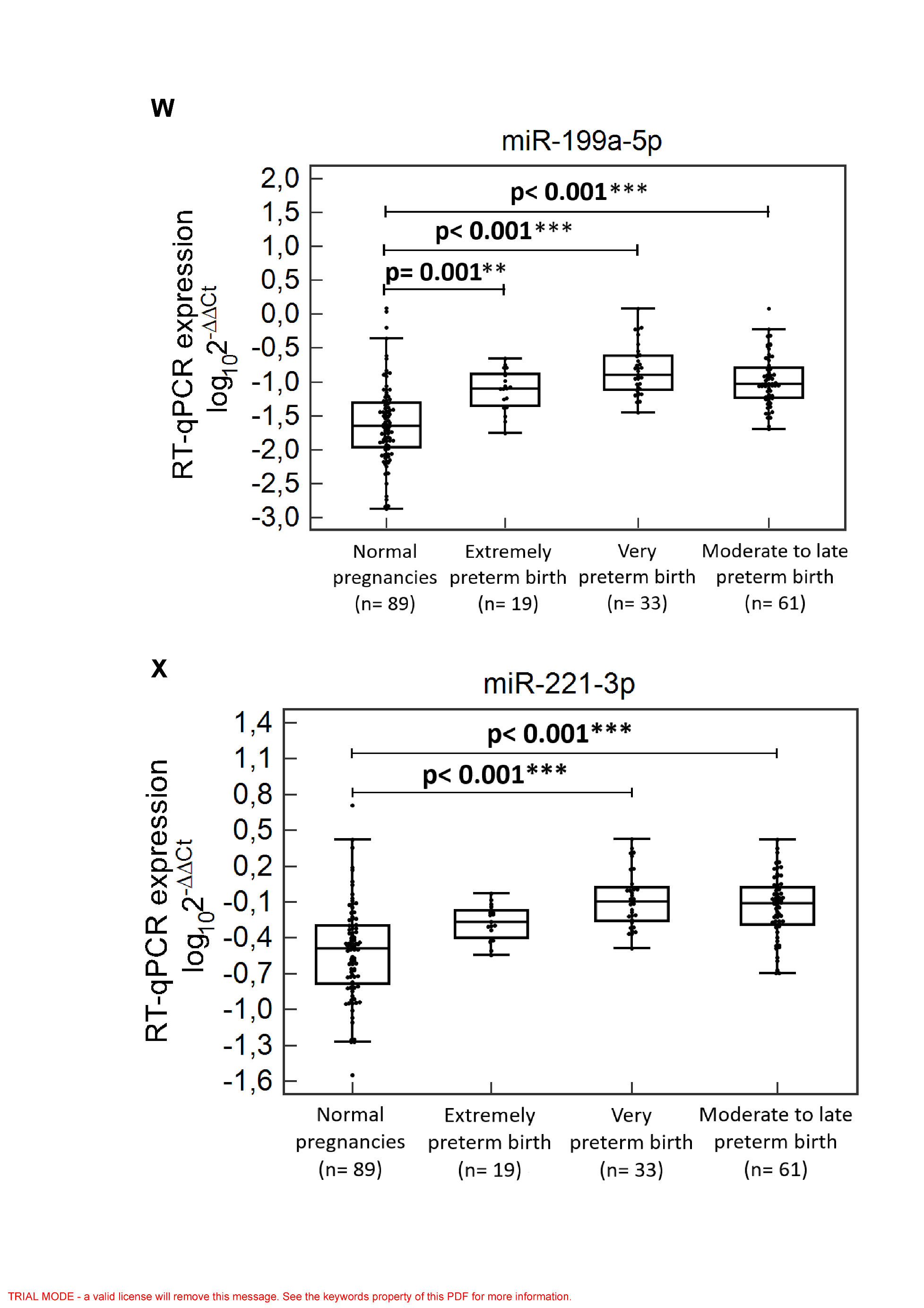


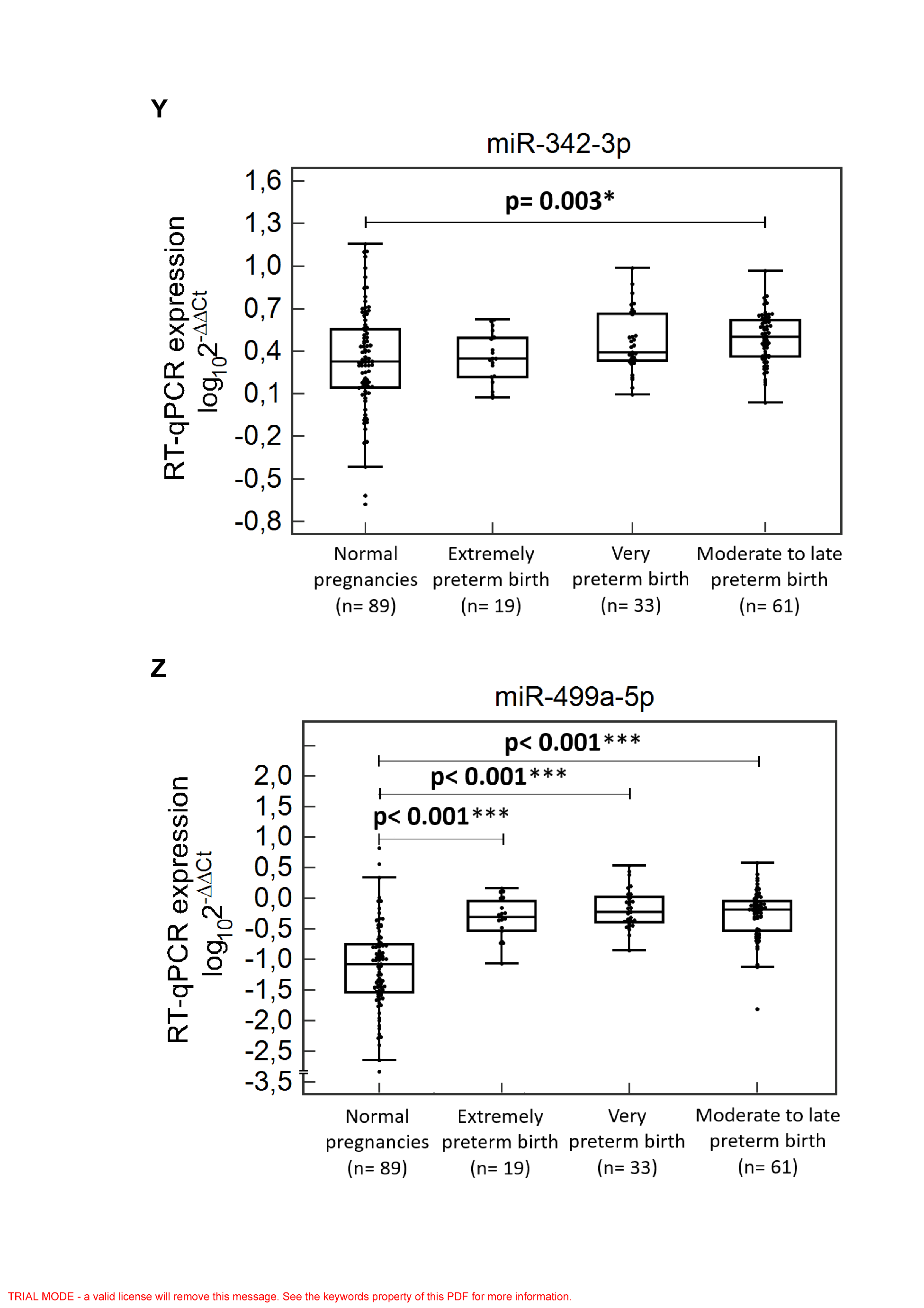


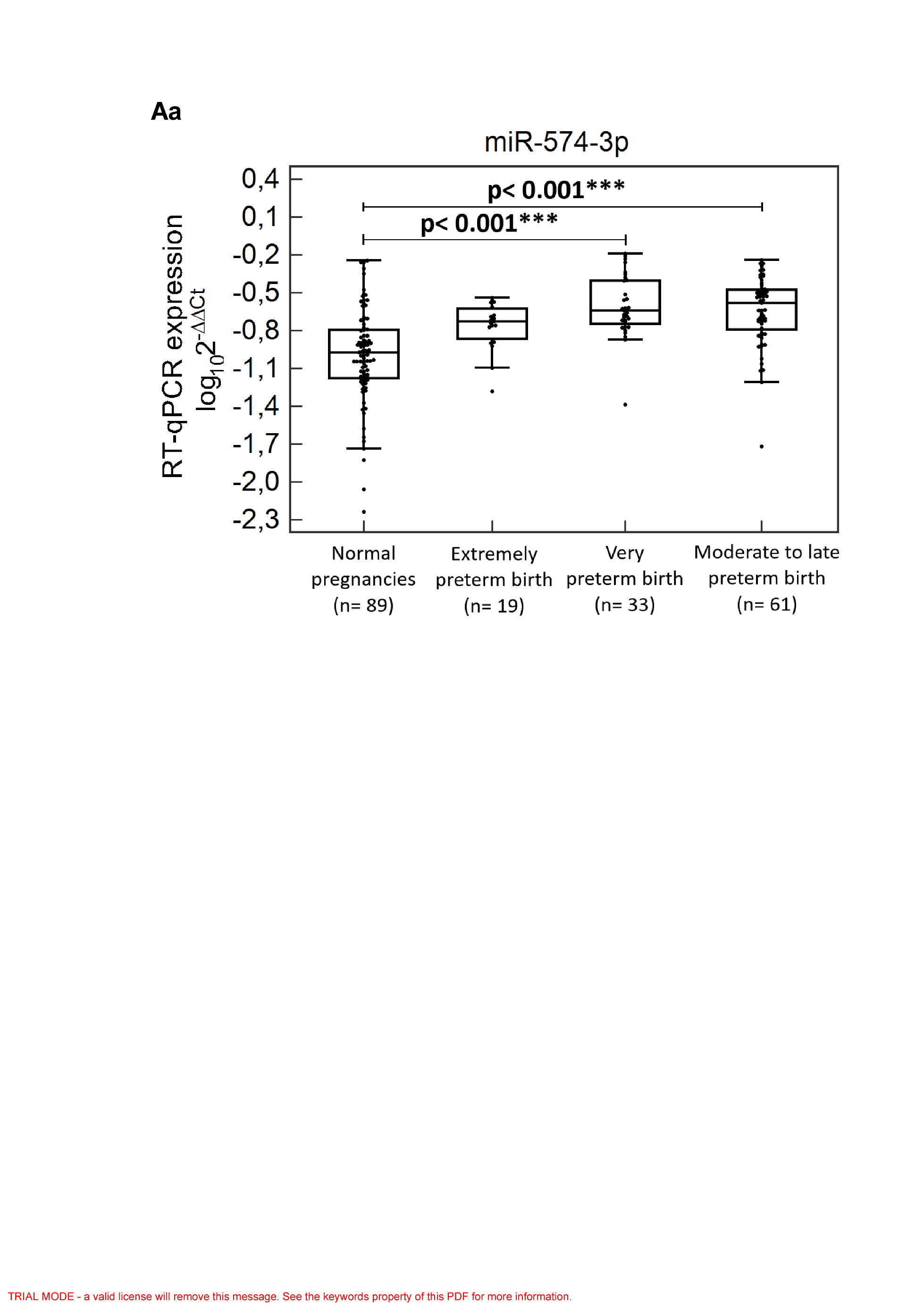


**Figure S2:** Postpartal microRNA expression profile in mothers with a history of preterm birth divided into individual subcategories based on gestational age. Mothers with a history of extremely preterm birth, very preterm birth, and moderate to late preterm birth had substantially increased postpartal expression of miR-1-3p, miR-20a-5p, miR-20b-5p, miR-26a-5p, miR-29a-3p, miR-100-5p, miR-103a-3p, miR-125b-5p, miR-126-3p, miR-130b-3p, miR-133a-3p, miR-143-3p, miR-145-5p, miR-181a-5p, miR-195-5p, miR-199a-5p, and miR-499a-5p. In addition, mothers with a history of very preterm birth and moderate to late preterm birth had substantially increased postpartal expression of miR-16-5p, miR-17-5p, miR-21-5p, miR-23a-3p, miR-24-3p, miR-146a-5p, miR-221-3p, and miR-574-3p. Besides, mothers with a history of moderate to late preterm birth had substantially increased postpartal expression of miR-342-3p. The Benjamini-Hochberg correction for multiple comparisons after Kruskal-Wallis test was applied to set up new cut-off values and interpret the experimental data (Table 3b). Significant results after the Benjamini-Hochberg correction are marked by * for α=0.05, ** for α=0.01, and *** for α=0.001.
