# Supplementary material for: A History of Preterm Delivery Is Associated with Aberrant Postpartal MicroRNA Expression Profiles in Mothers with an Absence of Other Pregnancy-Related Complications
Source: Int J Mol Sci. 2021 Apr 14;22(8):4033. doi: 10.3390/ijms22084033 (PMC8070839; doi:10.3390/ijms22084033)
Supplement: Supplementary file 1 [file ijms-22-04033-s001.zip › Supplementary Material/Supplementary Figure S3.docx]

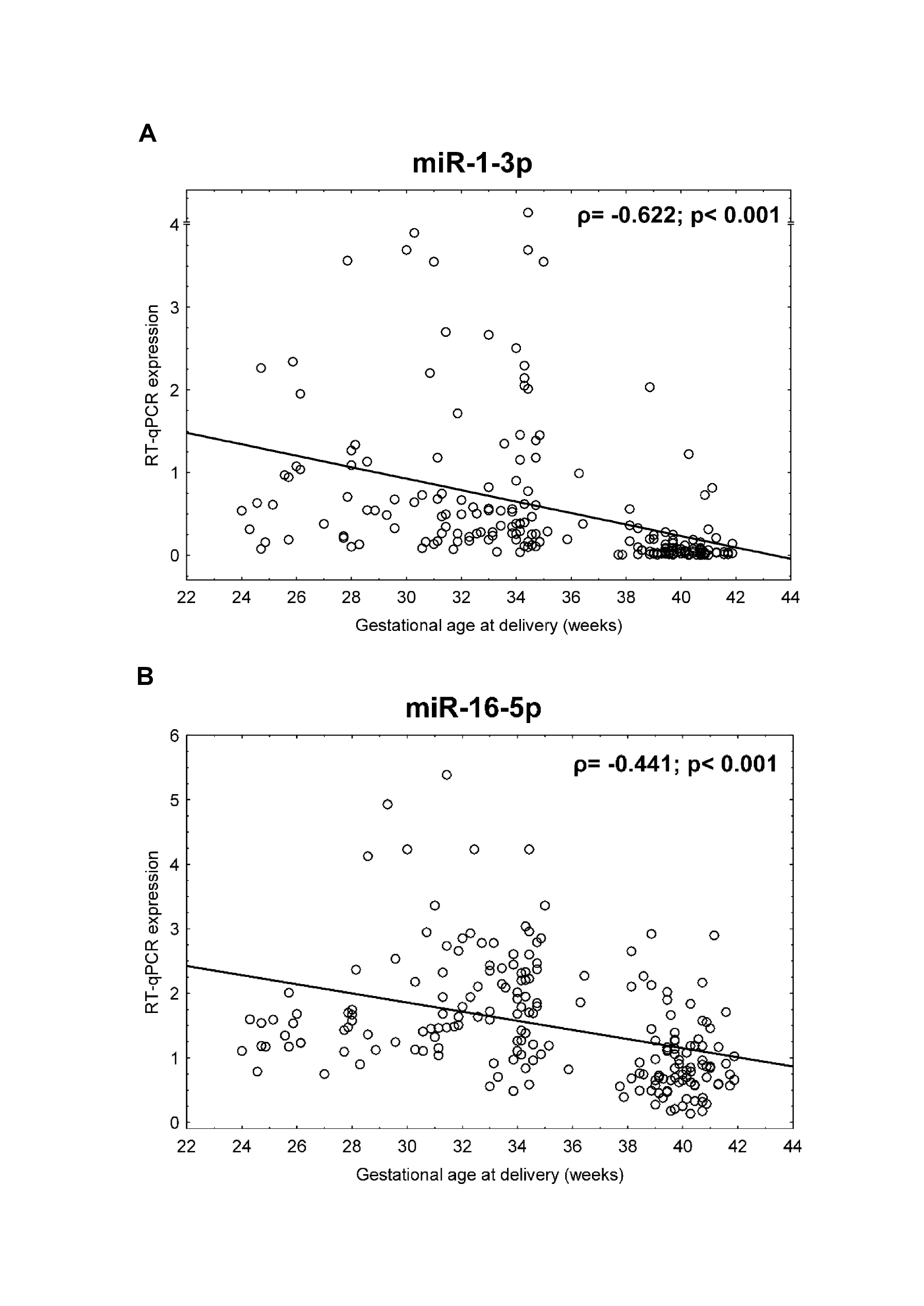
**Supplementary Figure S3.**


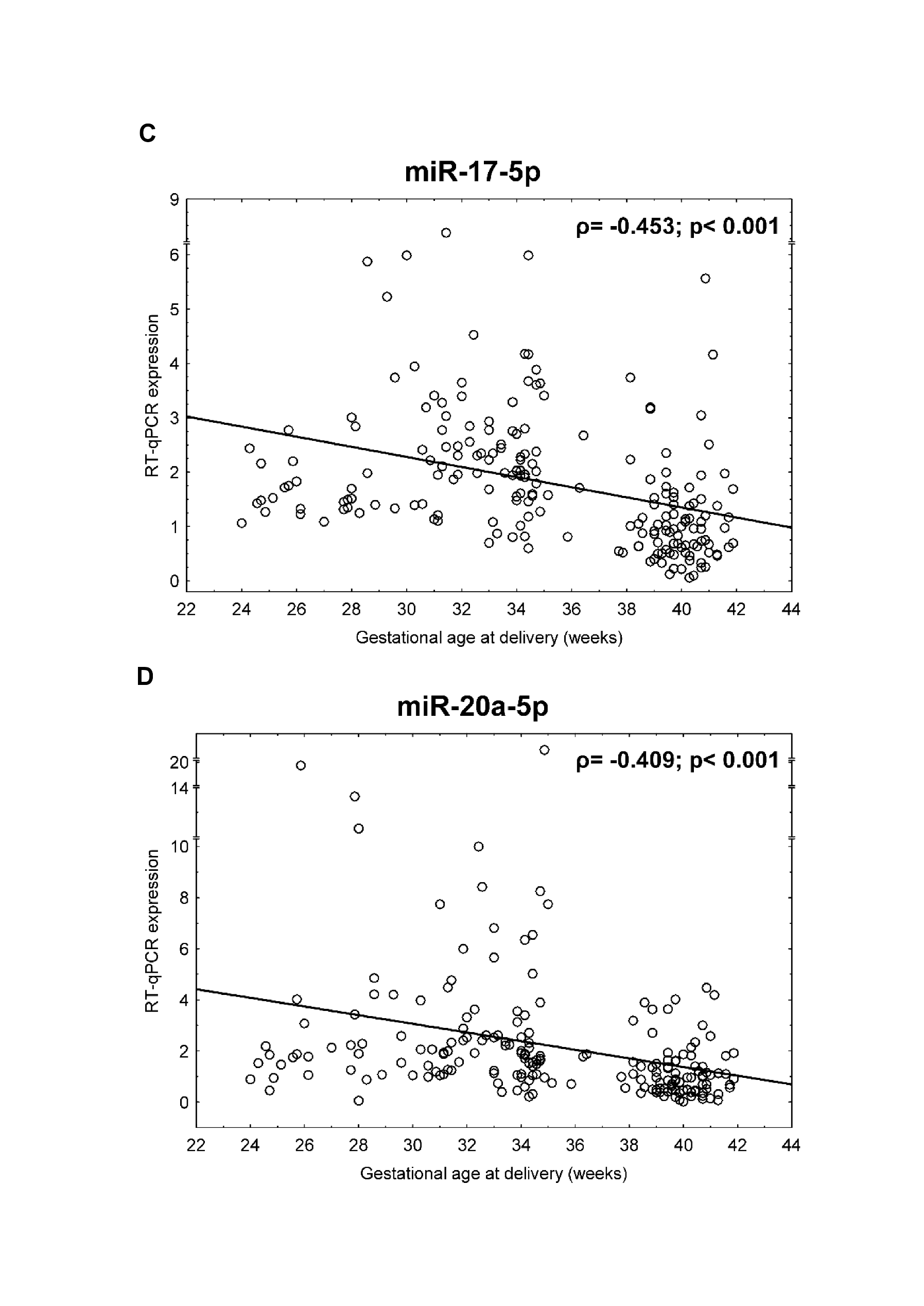


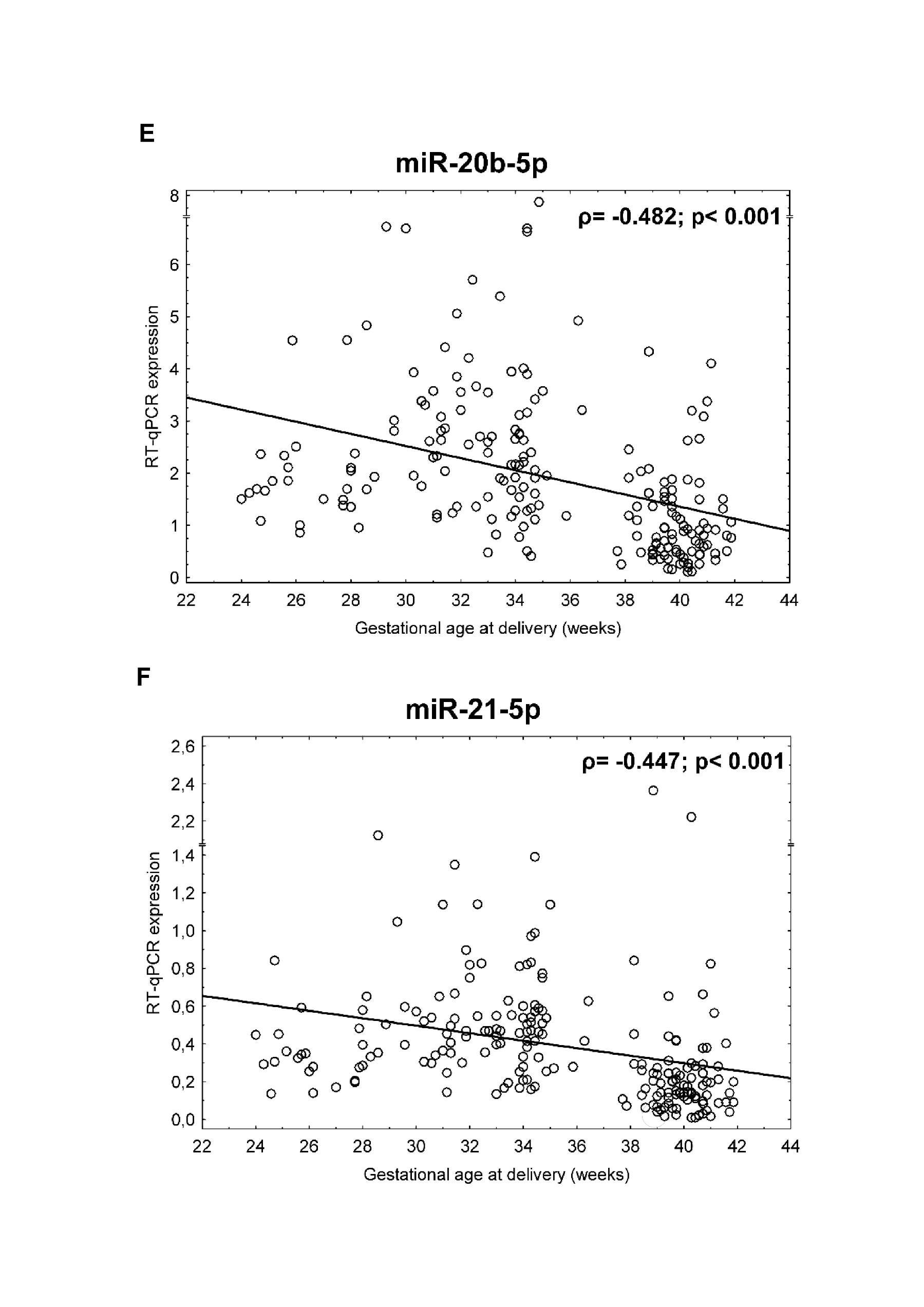


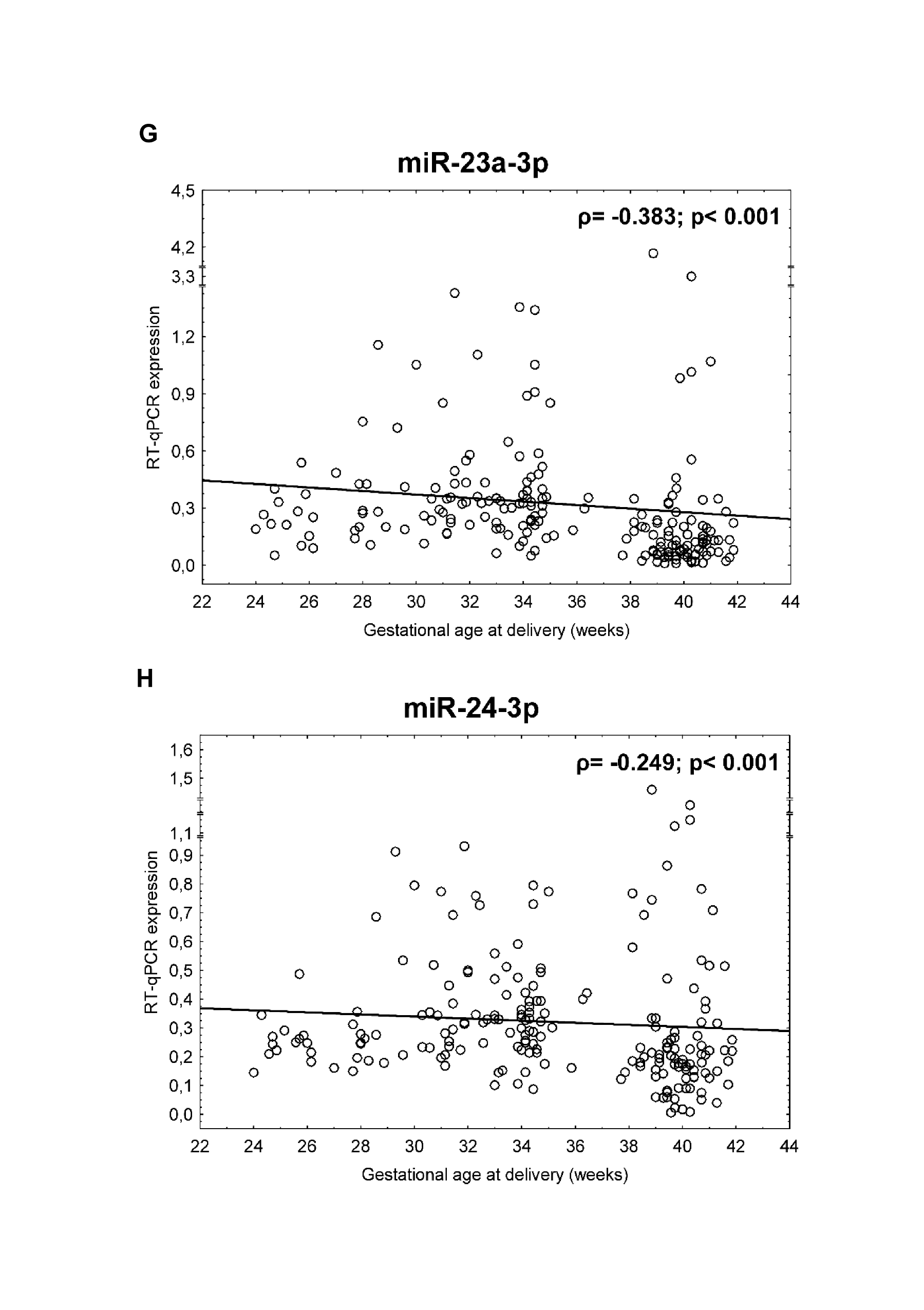


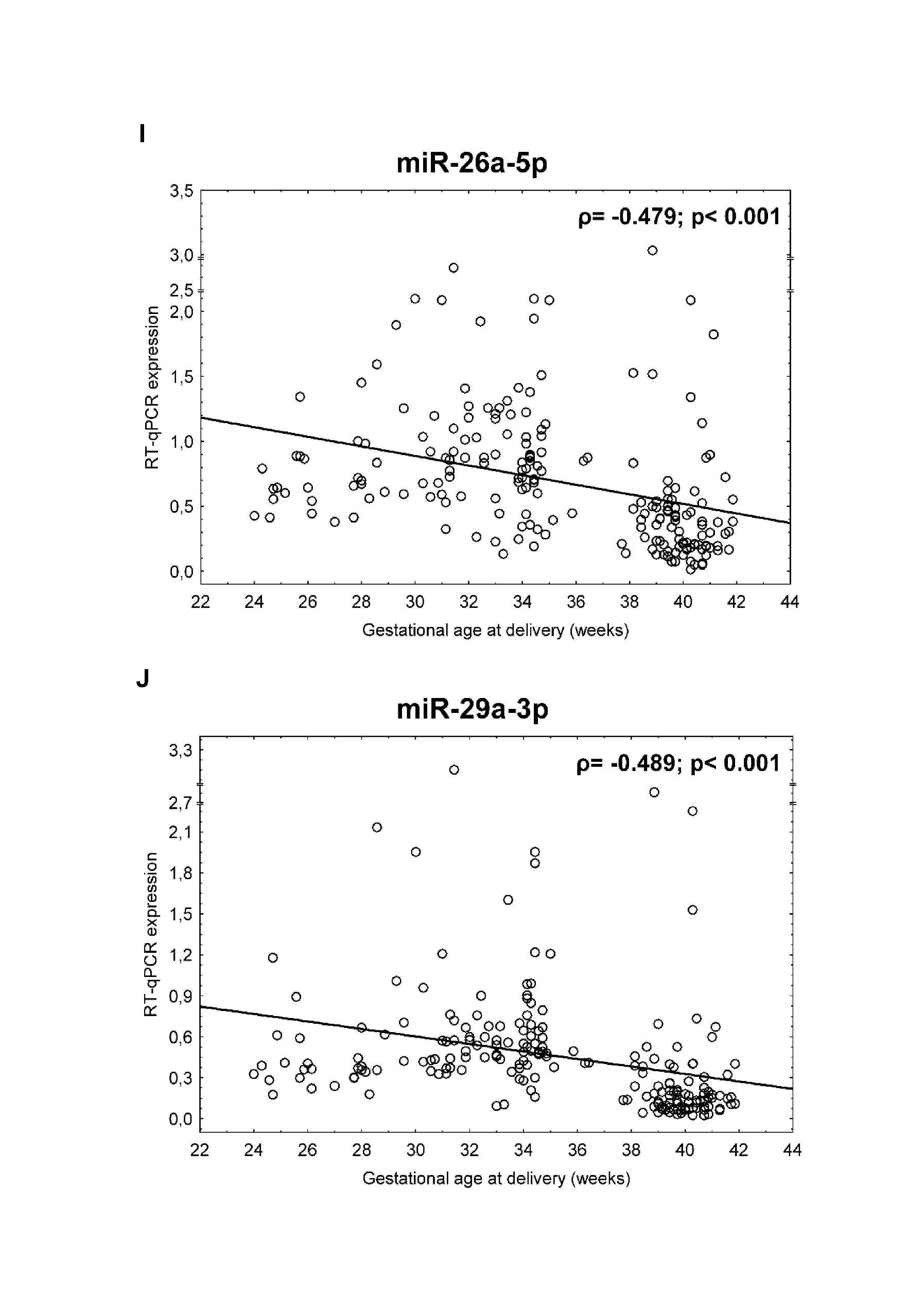


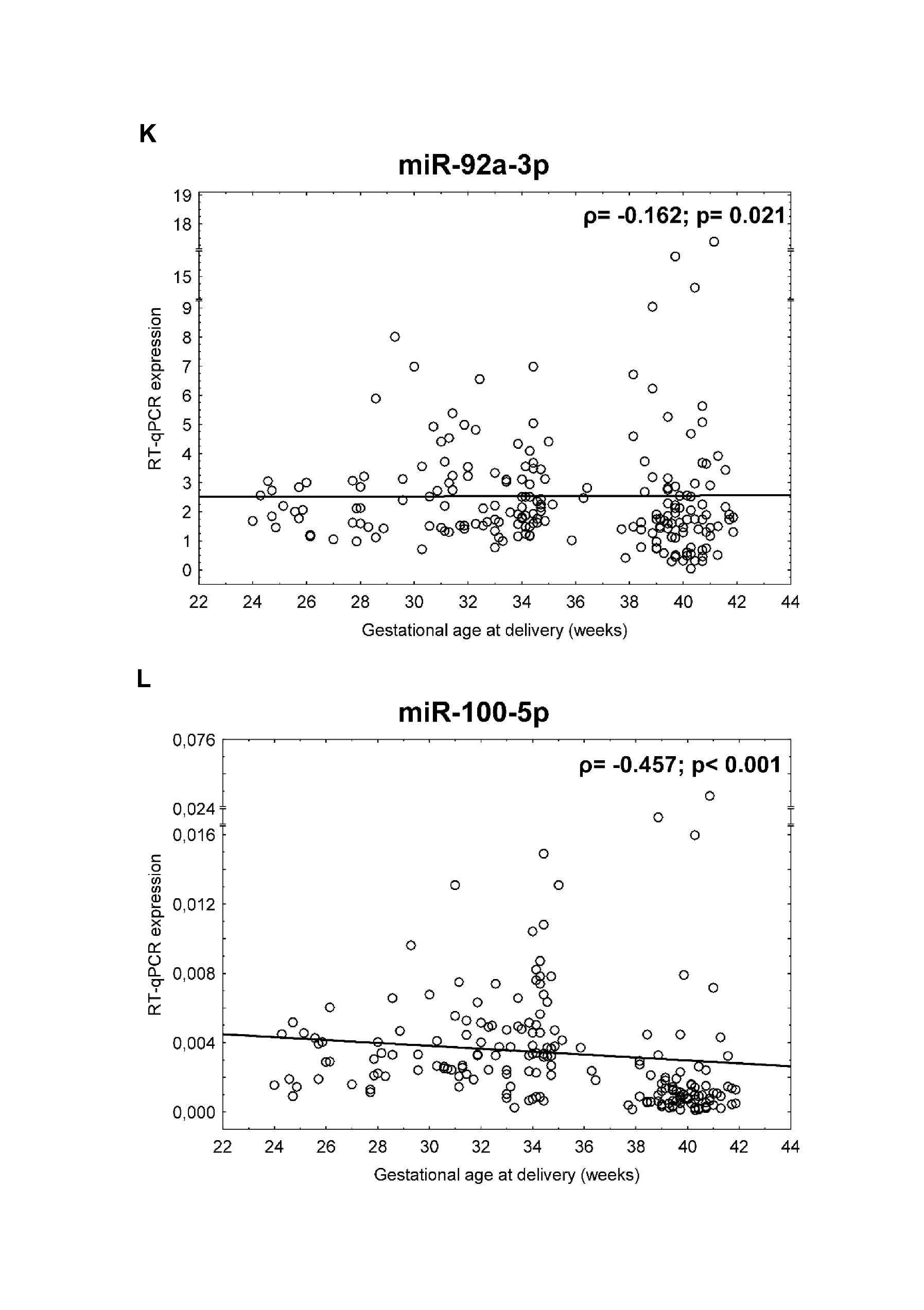


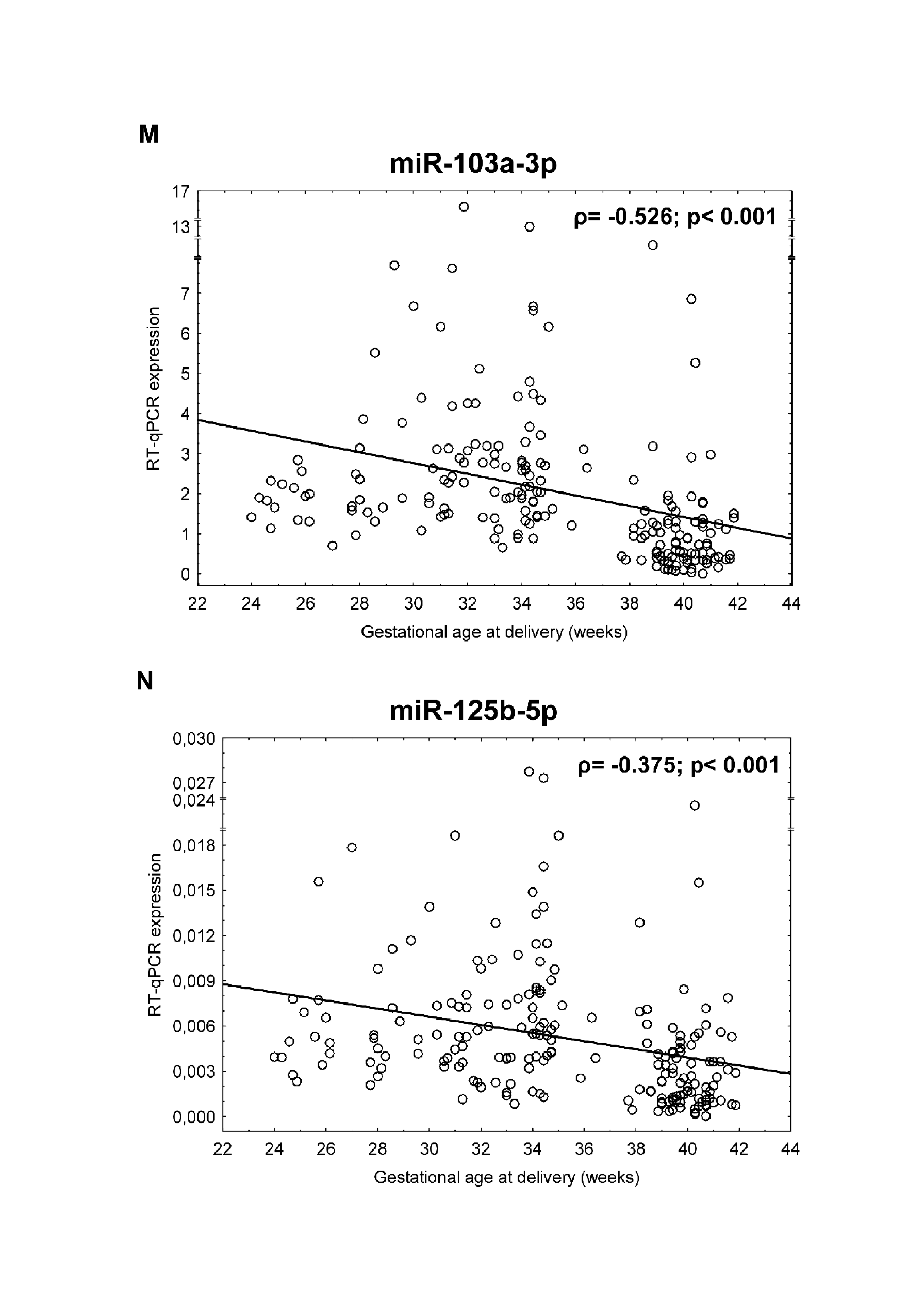


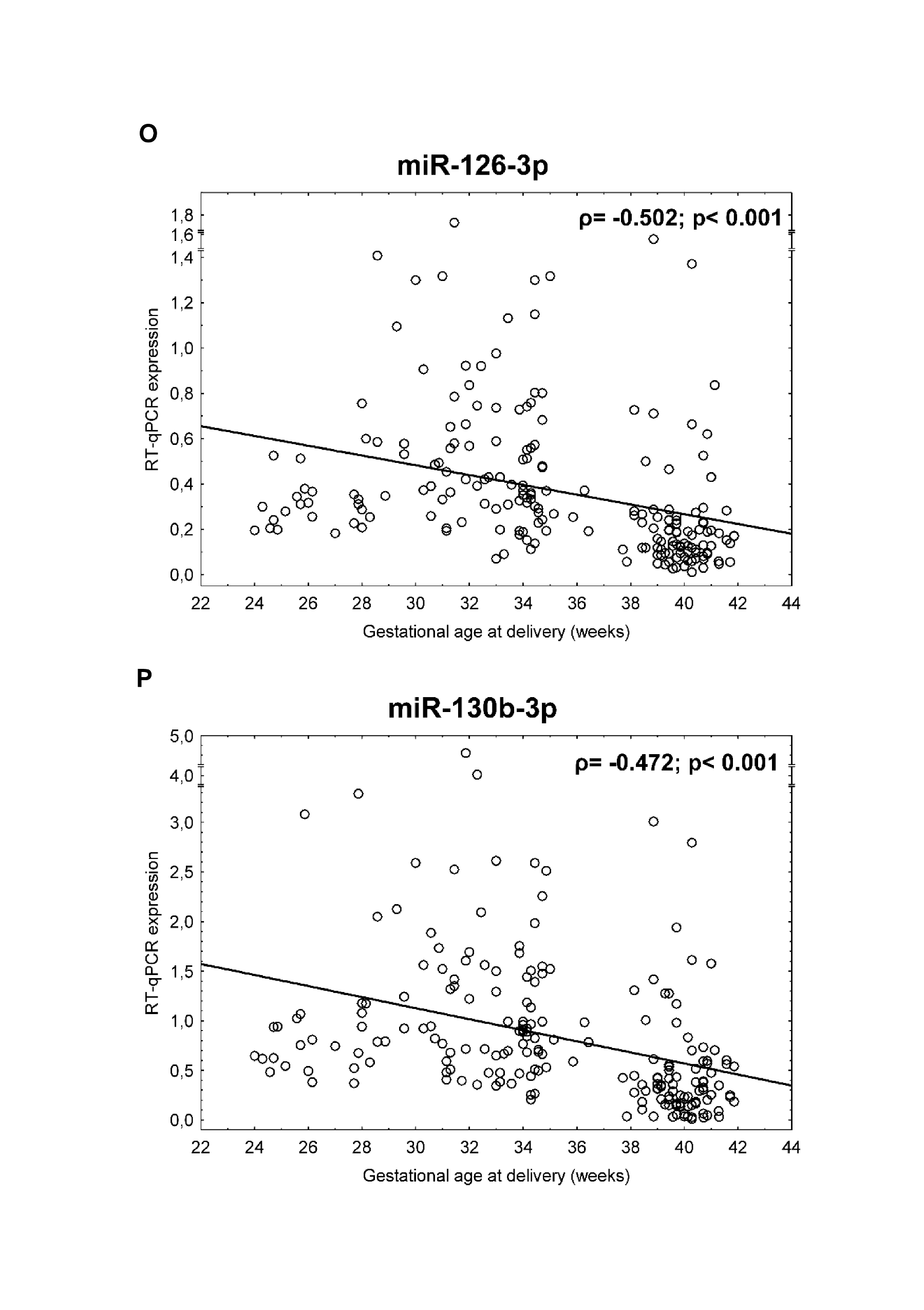


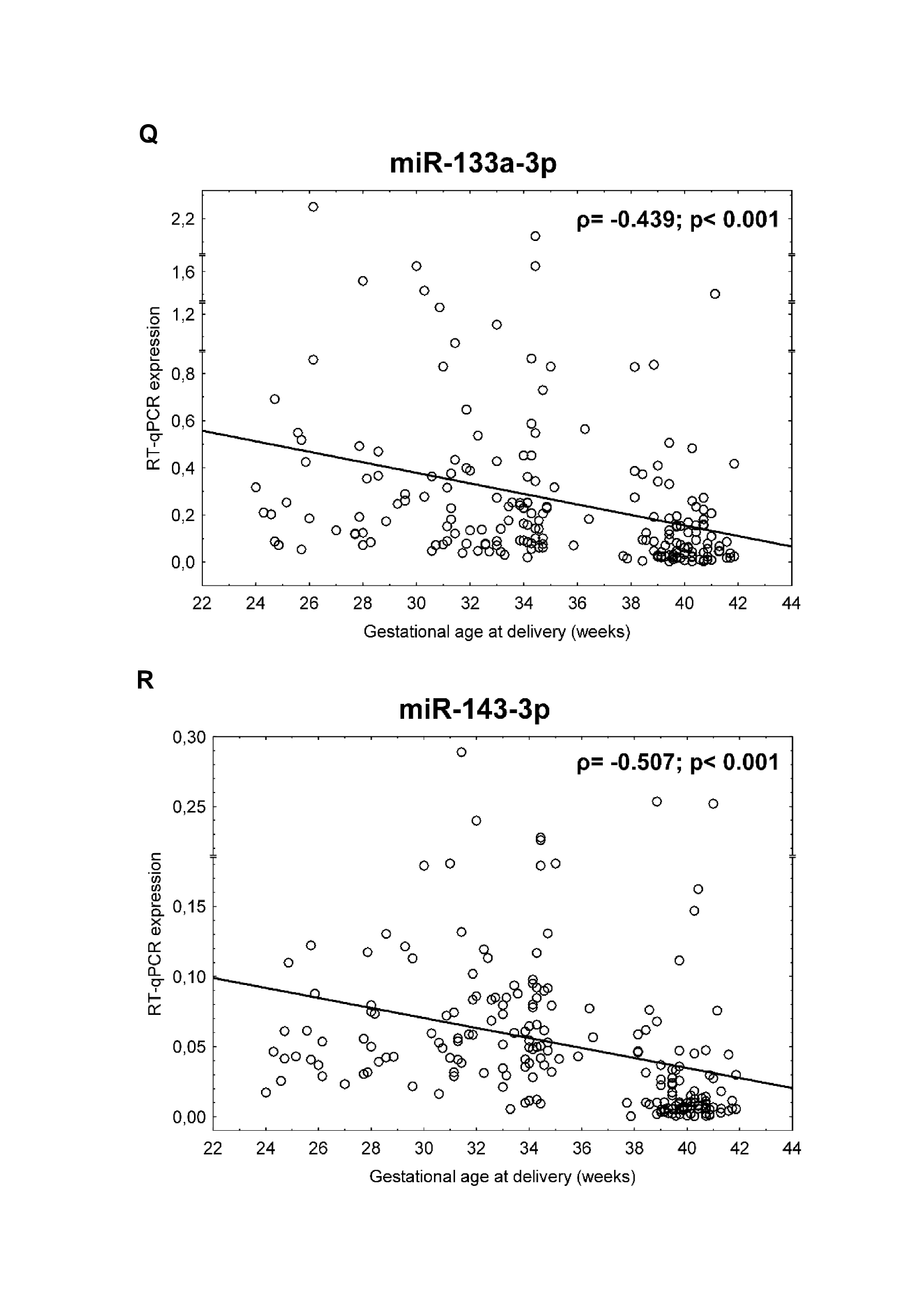


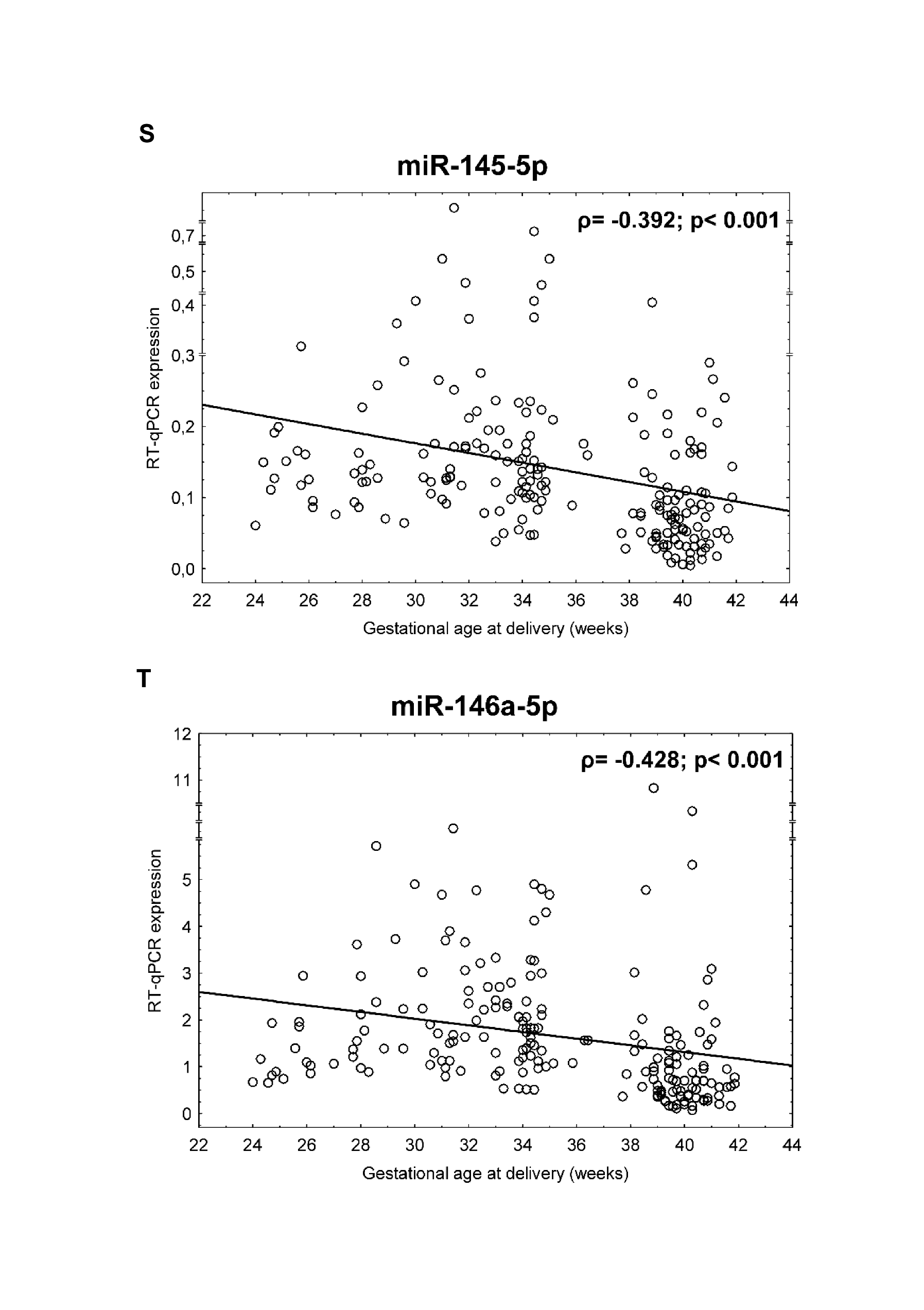


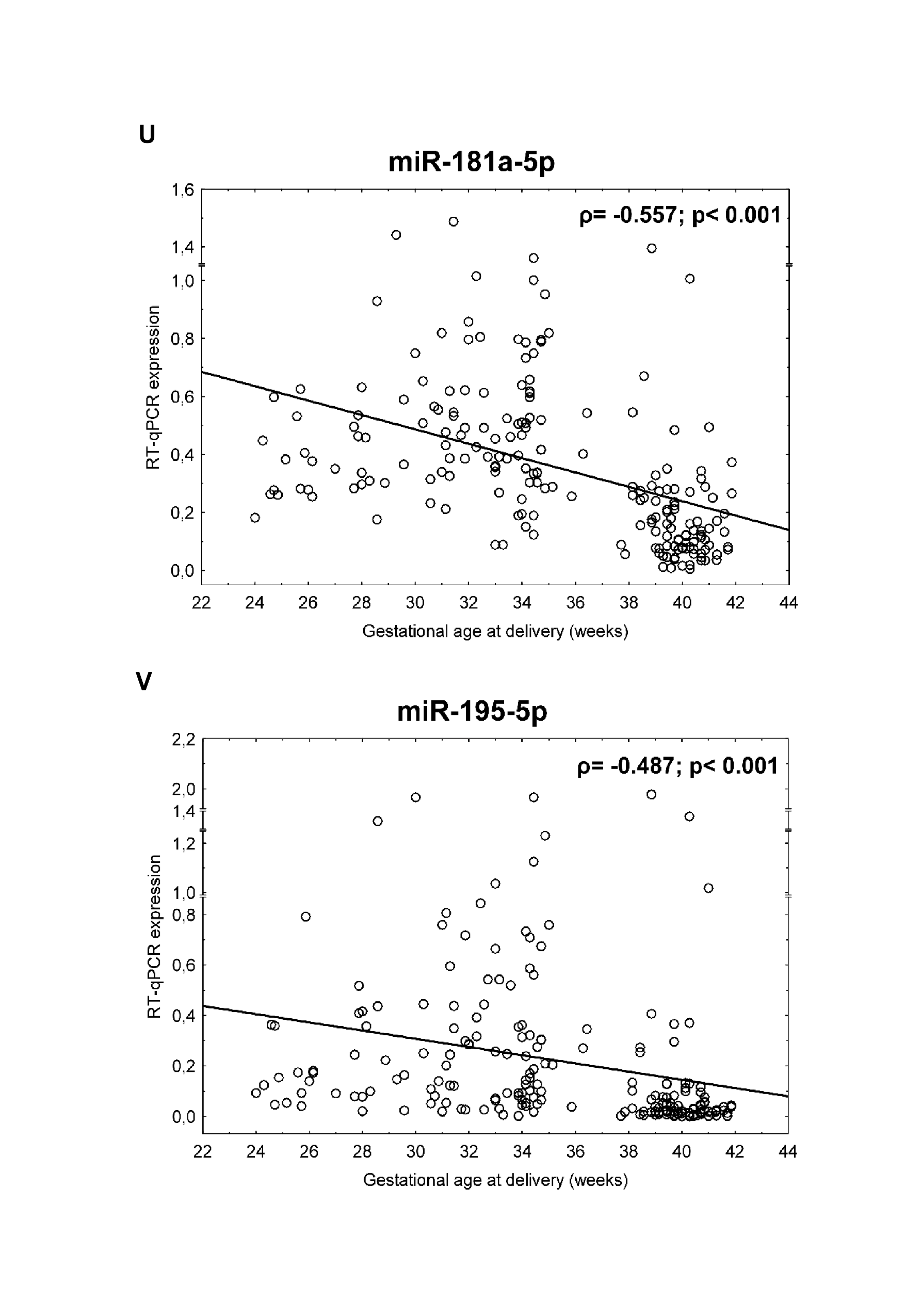


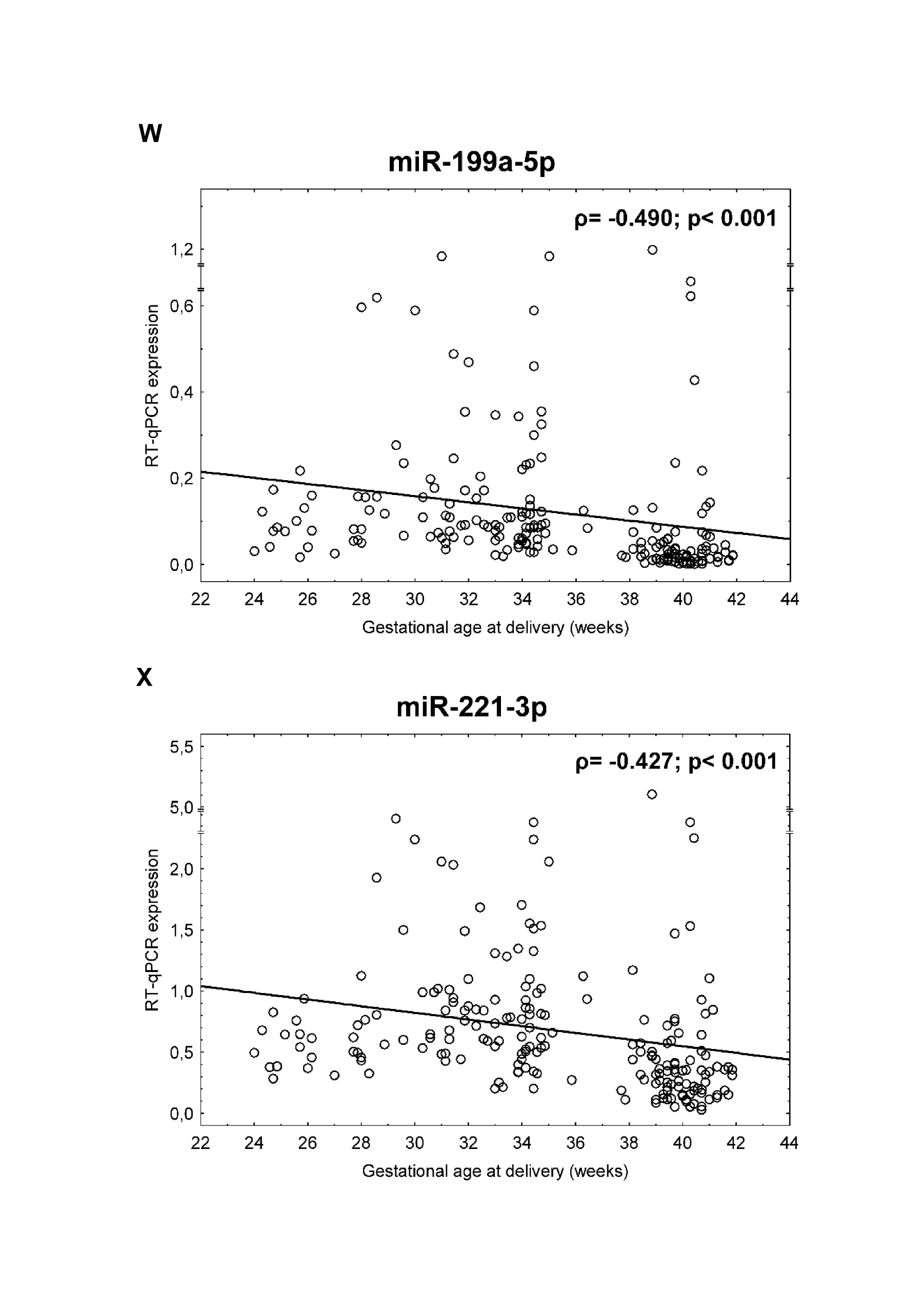


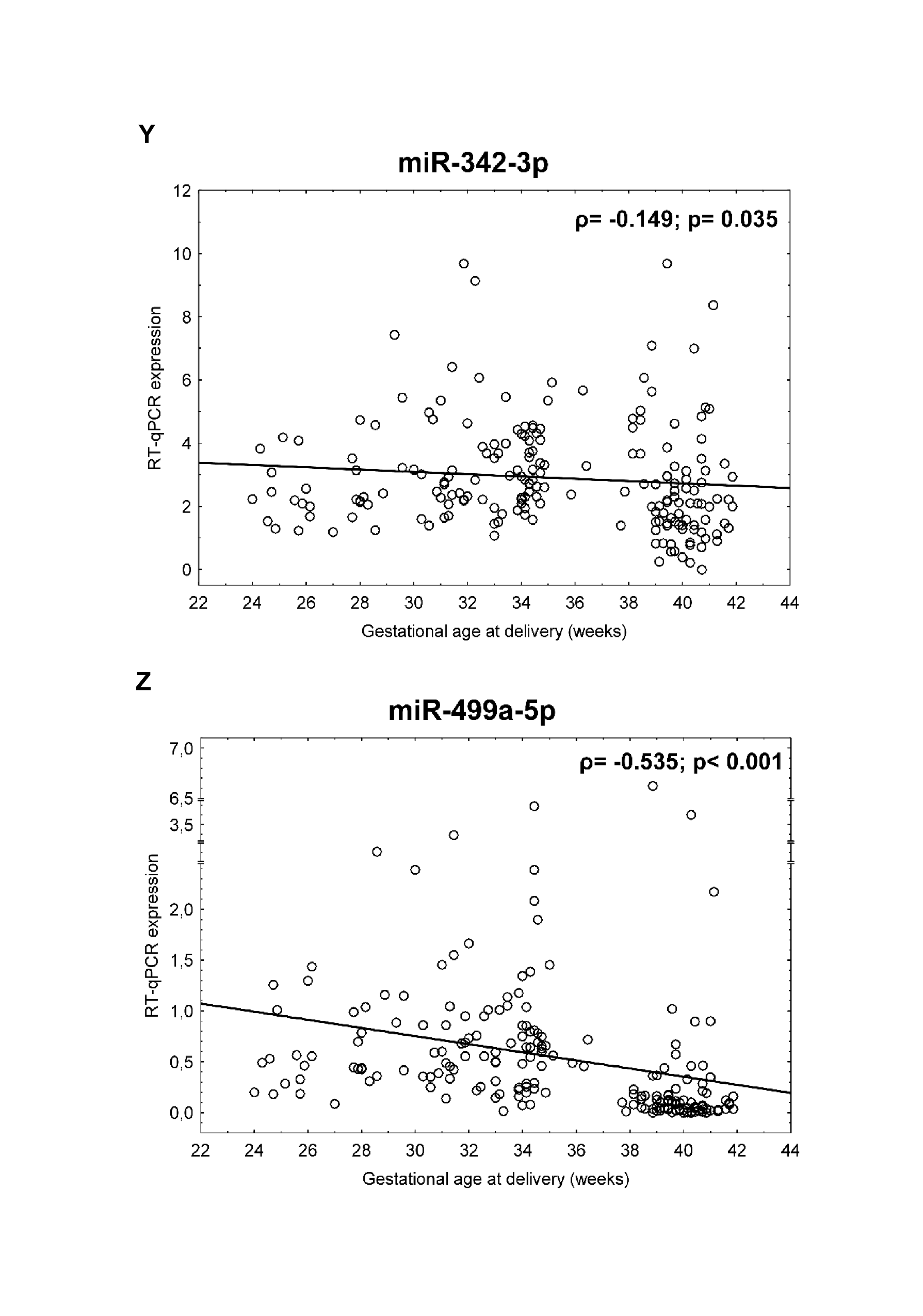


**
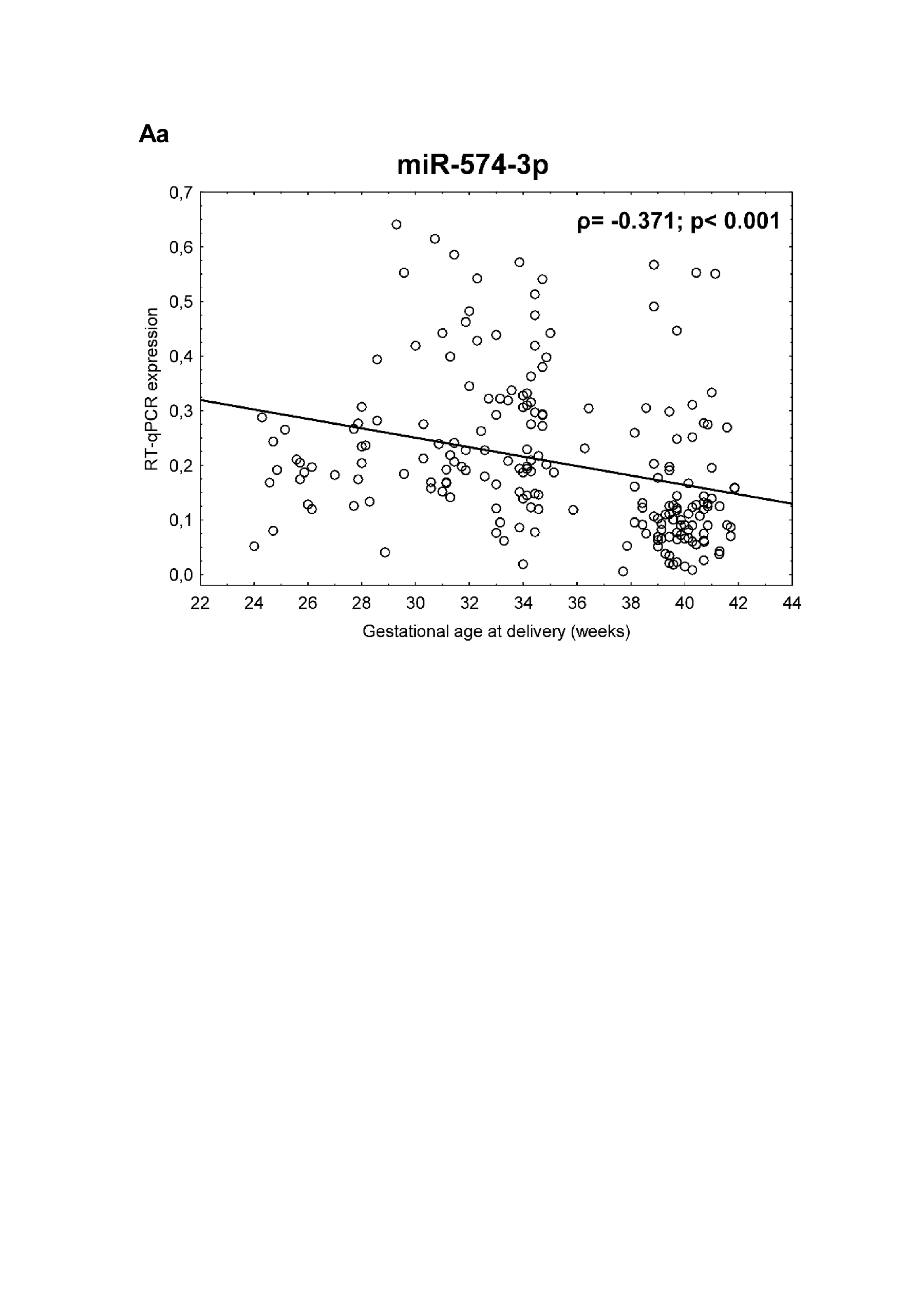
Figure S3:** Decline of postpartal microRNA gene expression in maternal peripheral white blood cells with advancing gestational age at the delivery.
