# Supplementary material for: A History of Preterm Delivery Is Associated with Aberrant Postpartal MicroRNA Expression Profiles in Mothers with an Absence of Other Pregnancy-Related Complications
Source: Int J Mol Sci. 2021 Apr 14;22(8):4033. doi: 10.3390/ijms22084033 (PMC8070839; doi:10.3390/ijms22084033)
Supplement: Supplementary file 1 [file ijms-22-04033-s001.zip › Supplementary Material/Supplementary Figure S5.docx]

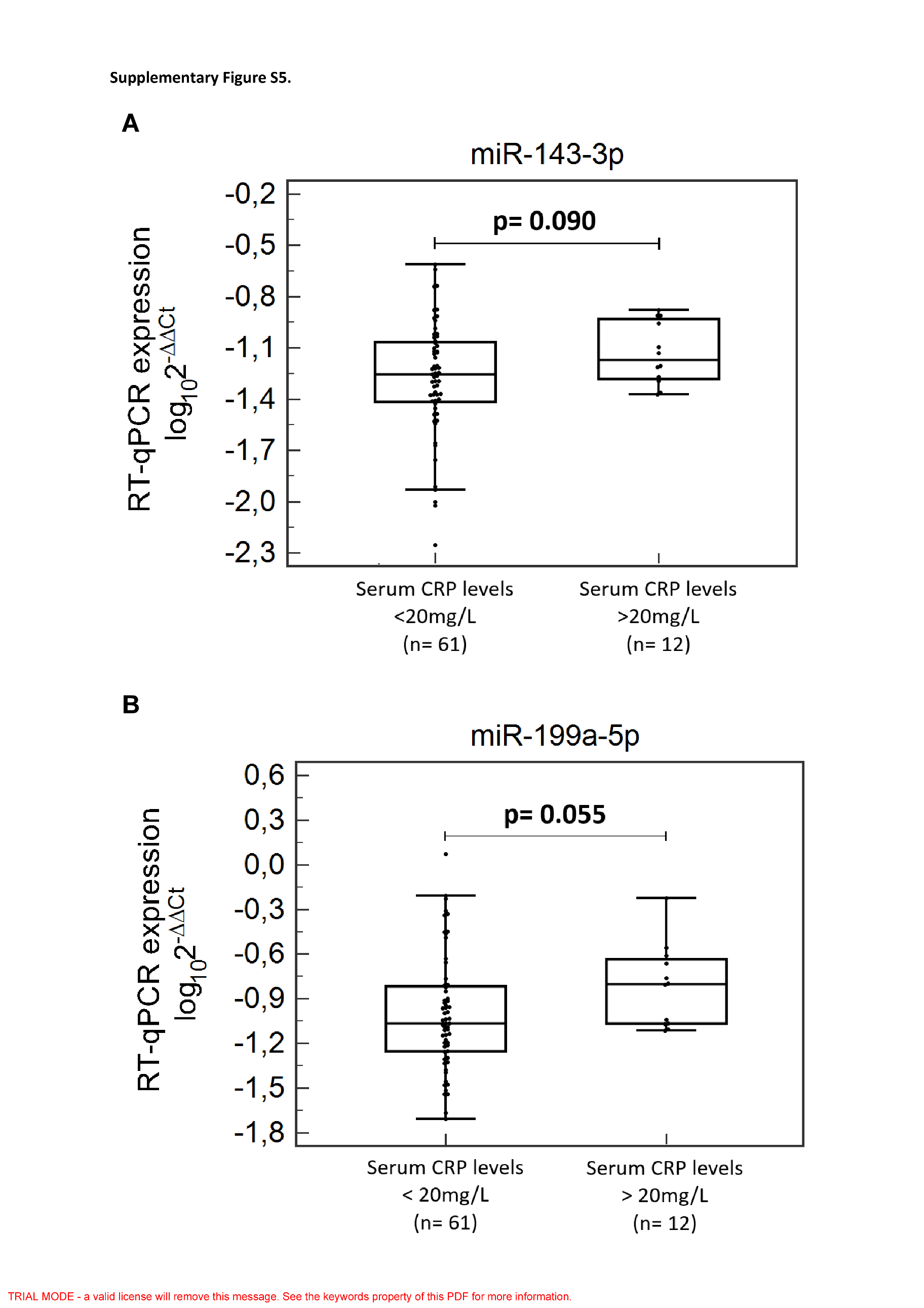


**Figure S5:** Postpartal microRNA expression profile in mothers with a history of preterm birth with relation to serum CRP levels during previous gestation. Mothers with a history of preterm birth, who had significantly increased serum CRP levels (above 20.0 mg/L) during previous gestation, showed a trend towards increased postpartal expression of miR-143-3p and miR-199a-5p.
