# Supplementary material for: A History of Preterm Delivery Is Associated with Aberrant Postpartal MicroRNA Expression Profiles in Mothers with an Absence of Other Pregnancy-Related Complications
Source: Int J Mol Sci. 2021 Apr 14;22(8):4033. doi: 10.3390/ijms22084033 (PMC8070839; doi:10.3390/ijms22084033)
Supplement: Supplementary file 1 [file ijms-22-04033-s001.zip › Supplementary Material/Supplementary Figure S6.docx]

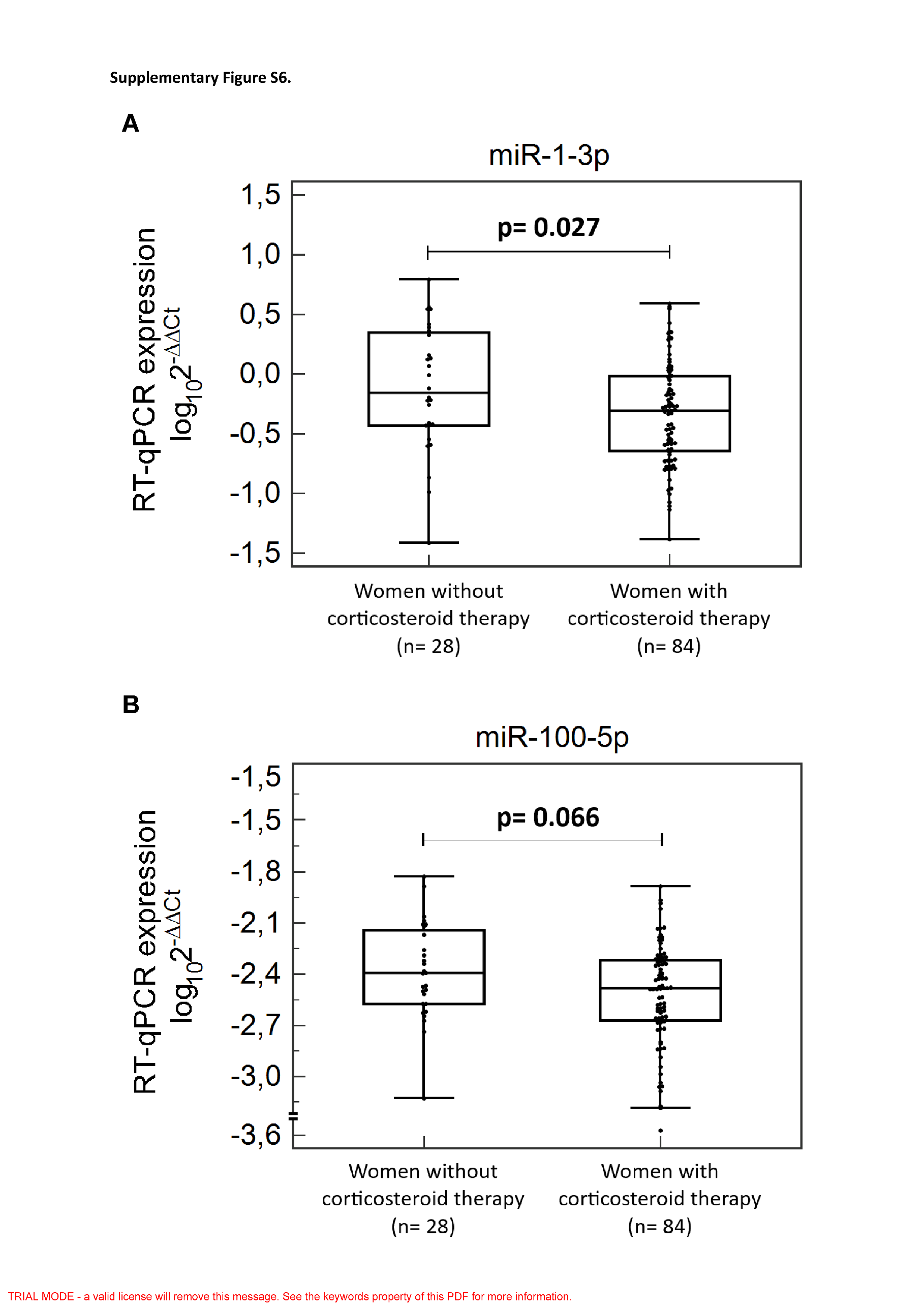


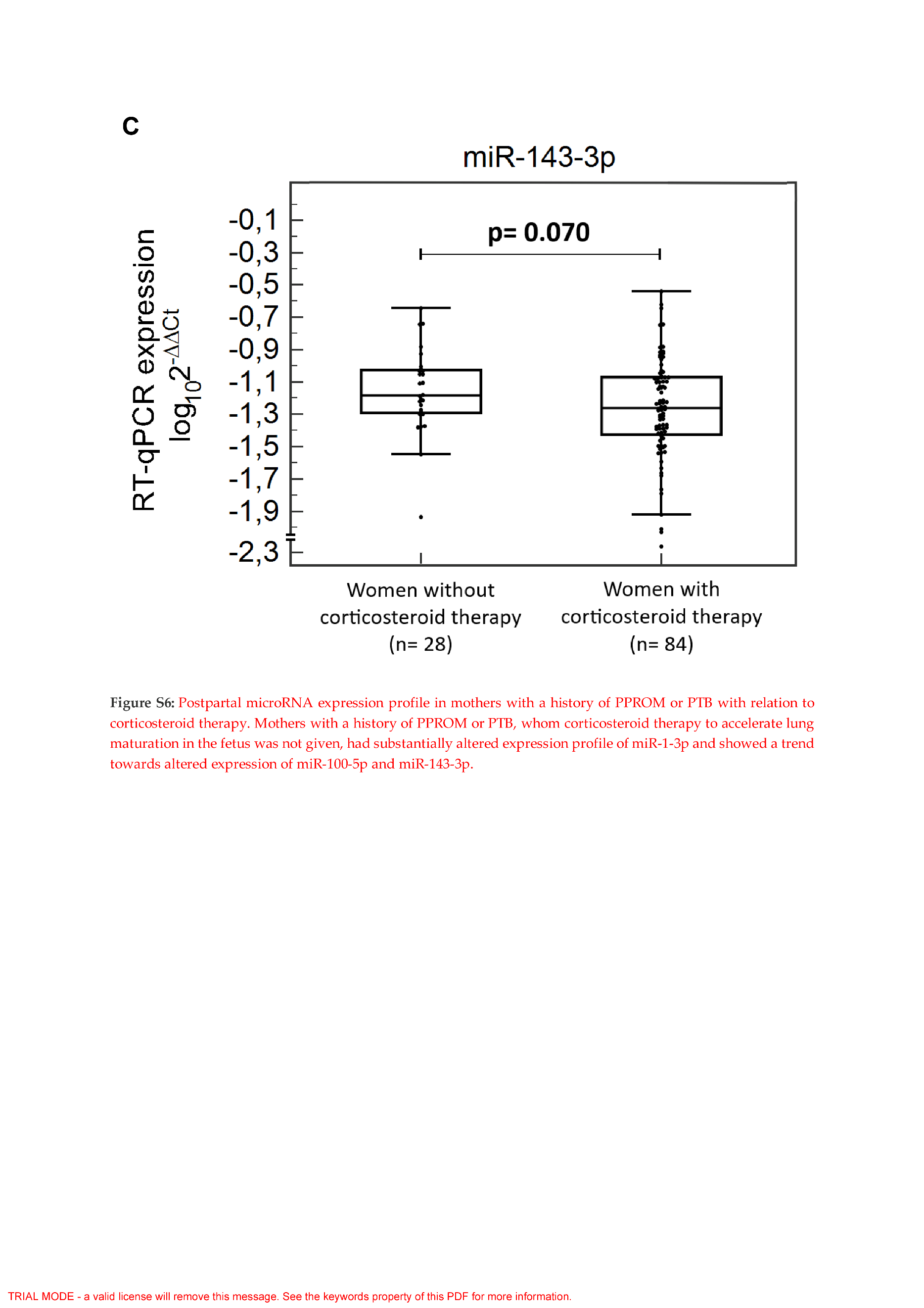


**Figure S6:** Postpartal microRNA expression profile in mothers with a history of PPROM or PTB with relation to corticosteroid therapy. Mothers with a history of PPROM or PTB, whom corticosteroid therapy to accelerate lung maturation in the fetus was not given, had substantially increased postpartal expression of miR-1-3p and showed a trend towards increased postpartal expression of miR-100-5p and miR-143-3p.
