# Supplementary material for: A History of Preterm Delivery Is Associated with Aberrant Postpartal MicroRNA Expression Profiles in Mothers with an Absence of Other Pregnancy-Related Complications
Source: Int J Mol Sci. 2021 Apr 14;22(8):4033. doi: 10.3390/ijms22084033 (PMC8070839; doi:10.3390/ijms22084033)
Supplement: Supplementary file 1 [file ijms-22-04033-s001.zip › Supplementary Material/Supplementary Figure S7.docx]

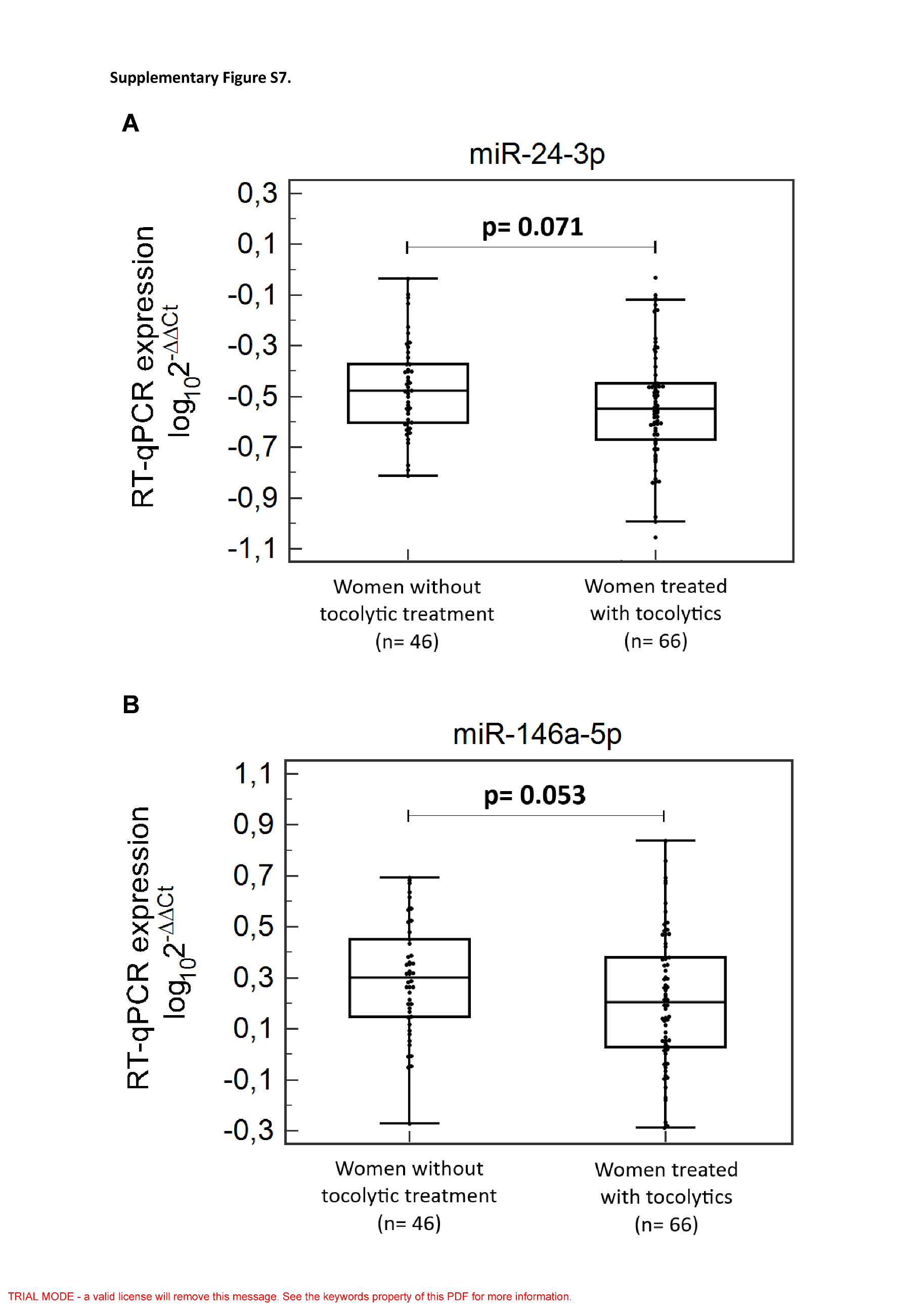


**Figure S7:** Postpartal microRNA expression profile in mothers with a history of PPROM or PTB with relation to tocolytic therapy. Mothers with a history of PPROM or PTB, whom tocolytic therapy to suppress premature labor was not given, showed a trend towards increased postpartal expression of miR-24-3p and miR-146a-5p.
