# Supplementary material for: A History of Preterm Delivery Is Associated with Aberrant Postpartal MicroRNA Expression Profiles in Mothers with an Absence of Other Pregnancy-Related Complications
Source: Int J Mol Sci. 2021 Apr 14;22(8):4033. doi: 10.3390/ijms22084033 (PMC8070839; doi:10.3390/ijms22084033)
Supplement: Supplementary file 1 [file ijms-22-04033-s001.zip › Supplementary Material/Supplementary Methods.docx]

Materials and Methods

1.1. Sample processing, reverse transcription, and relative microRNA quantification

Processing of samples, reverse transcription, and relative quantification of microRNAs was done as previously described [1, 2]. In brief, homogenized cell lysates were prepared from samples of unclotted whole peripheral venous blood (200 µl) as soon as possible after blood collection using QIAamp RNA Blood Mini Kit (Qiagen, Hilden, Germany, no: 52304).

Total RNA was isolated using a mirVana microRNA Isolation kit (Ambion, Austin, TX, USA, no: AM1560) and treated with DNase I (Thermo Fisher Scientific, Carlsbad, CA, USA, no: EN0521).

Reverse transcription was performed using microRNA-specific stem-loop RT primers, components of TaqMan MicroRNA Assays, and TaqMan MicroRNA Reverse Transcription Kit (Applied Biosystems, Branchburg, NJ, USA, no: 4366597) in a total reaction volume of 10 µL on a 7500 Real-Time PCR system (Applied Biosystems, Branchburg, NJ, USA) under the following conditions: 30 minutes at 16 °C, 30 minutes at 42 °C, 5 minutes at 85 °C, and then held at 4 °C.

Relative quantification of microRNAs was performed by using real-time PCR in a total reaction volume of 15 µL, in which case cDNA (3 µL) was mixed with specific TaqMan MGB primers and probes (TaqMan MicroRNA Assay, Applied Biosystems, Branchburg, NJ, USA), and the constituents of the TaqMan Universal PCR Master Mix (Applied Biosystems, Branchburg, NJ, USA, no: 4318157). The samples were regarded as positive if the amplification occurred at Ct <40 (Ct, threshold cycle).

The expression of studied microRNAs was determined using the comparative Ct method [3], and normalized to endogenous controls with the lowest expression variability between studied samples (geometric mean of RNU58A and RNU38B) [4]. A reference sample, fetal part of one randomly selected placenta of normally ongoing pregnancy, was used throughout the study for relative microRNA quantification.

1.2. Data processing

Since our experimental data did not show a normal distribution using the Shapiro-Wilk test [5], microRNA levels were compared among the appropriate groups using non-parametric tests (the Mann-Whitney test and Kruskal-Wallis test). In case of Mann-Whitney test *p-*value of *p* < 0.05 was considered as statistical significant.The Benjamini-Hochberg correction controlling the false discovery rate (FDR) using sequential modified [Bonferroni correction](https://doi.org/10.1007/978-1-4419-9863-7_1213) for multiple comparisons was applied after Kruskal-Wallis test to set up new cut-off values and interpret the experimental data (Table 5a, 5b).

Correlation between variables was calculated using the Spearman rank correlation coefficient (ρ). If the correlation coefficient values ranged within <-1.0;-0.5>, there was a strong negative correlation. If it was within the interval <-0.5; 0>, there was a weak negative correlation.

Box plots of log-normalized gene expression values (RT-qPCR expression, log_10_ 2^-ΔΔCt^) were generated for particular microRNAs using Statistica software (version 9.0; StatSoft, Inc., Tulsa, OK, USA). The box plots display the medians, the 75^th^ and 25^th^ percentiles (the upper and lower limits of the boxes), the maximum and minimum values (the upper and lower whiskers), outliers (circles), and extremes (asterisks). Dot plots, all observations, are also displayed in the charts.

References:

1. Hromadnikova, I.; Kotlabova, K.; Dvorakova, L.; Krofta, L. Postpartum profiling of microRNAs involved in pathogenesis of cardiovascular/cerebrovascular diseases in women exposed to pregnancy-related complications. *Int J Cardiol* **2019**, *291*, 158-167.
2. Hromadnikova, I.; Kotlabova, K.; Dvorakova, L.; Krofta, L. Diabetes Mellitus and Cardiovascular Risk Assessment in Mothers with a History of Gestational Diabetes Mellitus Based on Postpartal Expression Profile of MicroRNAs Associated with Diabetes Mellitus and Cardiovascular and Cerebrovascular Diseases. *Int J Mol Sci* **2020**, *21*, 2437.
3. Livak, K.J.; Schmittgen, T.D. Analysis of relative gene expression data using real-time quantitative PCR and the 2(-Delta Delta C(T)) Method. *Methods* **2001**, 25, 402–408.
4. Vandesompele, J.; de Preter, K.; Pattyn, F.; Poppe, B.; Van Roy, N.; de Paepe, A.; Speleman, F. Accurate normalization of real-time quantitative RT-PCR data by geometric averaging of multiple internal control genes. *Genome Biol* **2002**, *3*, research0034.
5. Shapiro, S.S.; Wilk, M.B. An Analysis of Variance Test for Normality (Complete Samples) *Biometrika* **1965**, *52*, 591–611.
